# Endosymbiotic bacteria within the nematode-trapping fungus *Arthrobotrys musiformis* and their potential roles in nitrogen cycling

**DOI:** 10.3389/fmicb.2024.1349447

**Published:** 2024-01-29

**Authors:** Hua Zheng, Tong Chen, Wenjie Li, Jianan Hong, Jianping Xu, Zefen Yu

**Affiliations:** ^1^Laboratory for Conservation and Utilization of Bio-Resources, Yunnan University, Kunming, China; ^2^School of Life Sciences, Yunnan University, Kunming, China; ^3^Department of Biology, McMaster University, Hamilton, ON, Canada

**Keywords:** nematode-trapping fungi, endosymbiosis, bacterial diversity, nitrogen metabolism, nitrogen-fixing bacteria, denitrifying bacteria, trap formation

## Abstract

Endosymbiotic bacteria (ESB) have important effects on their hosts, contributing to its growth, reproduction and biological functions. Although the effects of exogenous bacteria on the trap formation of nematode-trapping fungi (NTF) have been revealed, the effects of ESB on NTF remain unknown. In this study, we investigated the species diversity of ESB in the NTF *Arthrobotrys musiformis* using high-throughput sequencing and culture-dependent approaches, and compared bacterial profiles to assess the effects of strain source and culture media on *A. musiformis*. PICRUSt2 and FAPROTAX were used to predict bacterial function. Our study revealed that bacterial communities in *A. musiformis* displayed high diversity and heterogeneity, with Proteobacteria, Firmicutes, Bacteroidetes and Actinobacteria as the dominant phyla. The ESB between *A. musiformis* groups isolated from different habitats and cultured in the same medium were more similar to each other than the other groups isolated from the same habitat but cultured in different media. Function analysis predicted a broad and diverse functional repertoire of ESB in *A. musiformis*, and unveiled that ESB have the potential to function in five modules of the nitrogen metabolism. We isolated nitrogen-fixing and denitrifying bacteria from the ESB and demonstrated their effects on trap formation of *A. musiformis*. Among seven bacteria that we tested, three bacterial species *Bacillus licheniformis*, *Achromobacter xylosoxidans* and *Stenotrophomonas maltophilia* were found to be efficient in inducing trap formation. In conclusion, this study revealed extensive ESB diversity within NTF and demonstrated that these bacteria likely play important roles in nitrogen cycling, including nematode trap formation.

## 1 Introduction

Endosymbiotic bacteria (ESB) refer to bacterial symbionts of fungi that reside within fungal hyphae and reproductive structure (Lumini et al., 2007). ESB and their fungal hosts can be highly mutually beneficial in their growth and reproduction, performing various biological and ecological functions and influencing many aspects of agriculture, forestry and medicine. For example, fungi and their ESB can interact synergistically to stimulate plant growth. The fungal endophyte *Pestalotiopsis* spp. produced the plant-growth promoting hormone indole-3-acetic acid (IAA), and *in vitro* production of IAA was enhanced by the presence of its endosymbiotic bacterium *Luteibacter* sp. (Hoffman et al., 2013). On the other hand, toxin-producing ESB contribute to fungal virulence. In the early 1980s, rhizoxin, which causes rice blight, was originally isolated from the saprotrophic fungus *Rhizopus microsporus* (Sato et al., 1983; Iwasaki et al., 1984), however, later studies found that rhizoxin was not synthesized by the fungus itself but by its endosymbiotic bacteria (Partida-Martinez and Hertweck, 2005; Scherlach et al., 2006).

Fungal ESB are best known from species in phyla Glomeromycota and Mucoromycotina (Bianciotto et al., 2003; Bertaux et al., 2005; Sharma et al., 2008; Sato et al., 2010; Fujimura et al., 2014). The knowledge of associations between fungal hosts and their ESB has steadily increased in recent years, and these studies showed that the capacity to harbor ESB is widespread among fungi (Barbieri et al., 2000, 2007; Bertaux et al., 2003, 2005; Sharma et al., 2008). Hoffman and Arnold (2010) examined 414 isolates of endophytic fungi collected in the Americas in 2010, representing four of the most species-rich classes of Ascomycota (Pezizomycetes, Sordariomycetes, Dothideomycetes, and Eurotiomycetes), for the presence of ESB using microscopy and molecular techniques. Their study provided a strong evidence that the ability of fungi to harbor ESB is widespread. More recently, researchers screened potential bacterial associates in over 700 phylogenetically diverse fungal isolates, including those from several previously unexplored phyla (Blastocladiomycota, Chytridiomycota, and Zoopagomycota), and found that bacterial associations with diverse fungal hosts appear to be the rule rather than the exception (Robinson et al., 2021). Results from these studies suggested that ESB deserve greater consideration in microbiome studies, and that future investigations into the diverse nature of fungal-bacterial associates will need to tailor methods to avoid affecting the natural communities associated with fungi.

Nematode-trapping fungi (NTF) are a special ecological group that can develop sophisticated trapping structures to capture, kill, and digest nematodes as food sources (Schmidt et al., 2007; Liu et al., 2009). According to trapping structures, NTF have been categorized into several types such as producers of constricting rings and adhesive traps including adhesive nets, columns, knobs, and non-constricting rings (Nordbring-Hertz et al., 2006, 2011). NTF are widely distributed around the world, and can grow as saprophytes in soils while entering the parasitic stage by developing specific traps. Such features have made NTF ideal biocontrol candidates against parasitic nematodes of plants and animals (Mankau, 1980; Jatala, 1986; Yang et al., 2011). Indeed, there have been significant research on developing NTF into commercial biocontrol agents over the last two decades. However, most biocontrol researches on NTF have focused on investigating their species diversity and abundance in different regions and habitats, taxonomy and phylogeny, and field experiments on nematode control (Gray, 1983; Mitsui, 1985; Jaffee et al., 1998; Jaffee, 2004). In recent years, more attention has been paid to the molecular mechanisms related to trap formation and pathogenicity, the origin and evolution of trapping structures, and proteomic changes during trap formation (Yang et al., 2007, 2011, 2012; Hsueh et al., 2013; Meerupati et al., 2013; Liu et al., 2014; Li et al., 2015). Relatively speaking, the association between bacteria and NTF is less well studied, but its importance cannot be underestimated. For example, Rucker and Zachariah (1987) found that several species of bacteria (e.g., *Bacillus subtilis*, *Proteus vulgaris* and *Pseudomonas aeruginosa*) could influence trap formation of *Dactylaria brochopaga* and *Arthrobotrys conoides*. In 2011, Li et al. (2011) found that *Chryseobacterium* sp. isolated from agricultural soil could induce traps of *A. oligospora,* and the bacterial species had a similar effect on three other NTF. Later, Wang et al. (2014) also found that *Stenotrophomonas maltophilia* isolated from cow dung could stimulate the production of *A. oligospora* traps to kill nematodes.

*Arthrobotrys musiformis* is an important member of NTF, and can trap nematodes by its three-dimensional hyphal network (Nordbring-Hertz et al., 2011). Already in the 1930s, Linford used *A. musiformis* to control root-knot nematodes and had some success (Linford, 1937; Linford and Oliveira, 1938; Linford and Yap, 1938). In addition, this species has predatory activity against infective larvae of parasitic nematodes in the gastrointestinal tract of sheep (Graminha et al., 2005). Therefore, *A. musiformis* has been considered as a potential biological control agent for animal and plant parasites. Although researches on ESB in fungi greatly increased in recent years, little is known about ESB in NTF. To date, there are few reports on the effects of bacteria on NTF, primarily those from soil and cow dung (Li et al., 2011; Wang et al., 2014). A comprehensive understanding of the diversity and functions of ESB in NTF is highly desirable.

Here, we aimed to more comprehensively unravel the diversity and function of ESB in the NTF *A. musiformis*. For this purpose, we isolated many *A. musiformis* strains from diverse habitats, such as cold alpine soil and agricultural soil. Specifically, the objectives of this study were to (i) characterize the diversity, composition and core microbiome of ESB in *A. musiformis* using high-throughput sequencing and culture-dependent approaches, (ii) to compare the effects of habitats and culture media on ESB compositions of *A. musiformis*, (iii) to preliminarily reveal the potential function of these ESB, (iv) to investigate the effect of culturable ESB on the trap formation of *A. musiformis* using a co-culture method. Our results provide novel insights on the relationship and interaction between ESB and NTF.

## 2 Materials and methods

### 2.1 Isolation and identification of *Arthrobotrys musiformis*

Soil samples were collected from Yunnan province, including agricultural soil (Kunming and Heijing), moist cold alpine soil (Diqing), diggings soil (Gejiu), salty soil (Heijing), and forest soil (Yuxi and Xishuangbanna) (Supplementary Table S1). In addition, sediments from Dianchi Lake in Kunming and fruiting bodies of *Orbilia* spp. from Xishuangbanna Tropical Botanical Garden in Yunnan province were sampled (Supplementary Table S1). Strains of *A. musiformis* were isolated using the standard “sprinkle plate” method in which a small quantity of soil was placed on corn meal agar (CMA; 20 g cornmeal, 18 g agar, 1,000 mL distilled water) plates while the nematode *Caenorhabditis elegans* was simultaneously added to stimulate the growth of NTF. After incubation for 2–3 weeks, many spore clusters of *A. musiformis* appeared, and these spores were picked to potato dextrose agar (PDA; 200 g potato, 20 g dextrose, 18 g agar, 1,000 mL distilled water) plates using a sterilized toothpick under a BX51 microscope (Olympus Corporation, Tokyo, Japan). These obtained strains were examined morphologically again, such as the sizes of conidia and conidiophores, colony colors and trap structure, and were purified and cultured.

Genomic DNA of all purified *A. musiformis* strains was extracted from fresh cultures grown on PDA plates for 7 days, following the protocol of Turner et al. (1997). For the identification of *A. musiformis*, the internal transcribed spacer (ITS) region was amplified with the ITS5/ITS4 primers (White et al., 1990). Amplification was performed in a total of 25 μL reaction volume, which contained 12.5 μL 2× MasterMix (Tiangen Biotech, Beijing, China), 1.0 μL of each forward and reverse primer, 1.0 μL DNA template, and 9.5 μL ddH_2_O. PCR reactions were run in an Eppendorf Mastercycler (Eppendorf, Hamburg, Germany) following the PCR thermal cycle programs described by Su et al. (2016). Then, the obtained PCR products were purified using a commercial kit (Bioteke Biotechnology, Beijing, China) and sequenced using both the forward and reverse primers with a LI-COR 4000 L automatic sequencer, using a Thermo Sequenase-kit as described by Kindermann et al. (1998).

### 2.2 Detection of ESB

The presence or absence of bacteria in living hyphae were examined by using light microscopy after staining with SYTO9 (Invitrogen, California, United States). We treated fresh mycelia, which growing on PDA plate for 4 days, in 15 μL SYTO9 stain, and placed for 15 min in the dark at room temperature. Then, the stained mycelia were spread onto slides for microscopic observation and photography. A Leica DM4000B microscope (Leica Microsystems, Wetzlar, Germany) with a Luminera camera and 100-W mercury arc lamp was used for fluorescent imaging. Visible fluorescence of bacterial nucleic acids within living hyphae, which could be distinguished with fungal mitochondrial or nuclear DNA according to the size and motility as described by previous studies (Hoffman and Arnold, 2010; Arendt et al., 2016; Shaffer et al., 2016; Indu et al., 2021), provided evidence of viable endosymbiotic bacteria (Stewart and Deacon, 1995).

After visual examination, we directly screened the total genomic DNA from the fungal cultures for the bacterial 16S rRNA as described by Shaffer et al. (2016). PCR products were visualized using gel electrophoresis, and positive products were sequenced.

### 2.3 High-throughput sequencing of ESB from *Arthrobotrys musiformis*

Total 40 strains of *A. musiformis* representing two habitats were selected, including 20 from agricultural soils collected on Xishan Mountain in Kunming (XS) and 20 from moist cold alpine soils sampled at Baima Snow Mountain in Diqing (BM). Xishan soil samples represented the environment highly affected by human activity, while Baima soil samples were collected from a pristine environment at an altitude of 4,900 meters. Considering that different media may influence ESB composition within *A. musiformis*, we comprehensively investigated the profiles of ESB when cultured in nutrient-rich potato dextrose broth (PDB; 200 g potato, 20 g dextrose, 1,000 mL distilled water) and nutrient-poor corn meal broth (CMB; 20 g cornmeal, 1,000 mL distilled water).

To ensure that our strains of *A. musiformis* were free of bacterial contamination, we tested whether the surface of hyphae and the liquid broth of our strains contained bacteria using Olympus BX51 microscopes with bright-field imaging. We also observed the liquid shaker culture of *A. musiformis* strains each day to see whether the medium suddenly became cloudy. Usually, the contaminated bacteria will quickly multiply and make the medium cloudy. Additionally, *A. musiformis* samples were filtered to obtain mycelia after liquid culture for 5 days at 28°C with shaking at 180 rpm, and the filtrates were coated on LB plate to observe whether bacteria grew. The filtrates were used as negative controls in our sequencing. All samples were divided into four groups according to habitats (i.e., strain source) and culture media, grouped as BMp (strains isolated from BM and cultured in PDB medium), BMc (strains isolated from BM and cultured in CMB medium), XSp (strains isolated from XS and cultured in PDB medium), and XSc (strains isolated from XS and cultured in CMB medium). Samples DNA was extracted using the CTAB method according to manufacturer’s instructions. Then, the amplification and sequencing of the V3–V4 region of the 16S rRNA gene were outsourced to LC-Biotechnology Corporation (Zhejiang, China), which were performed on NovaSeq PE250 platform. The primers and procedures of PCR amplification and sequencing were as described by Wang et al. (2023). All generated raw data in this study was deposited in the National Center for Biotechnology Information Sequence Read Archives (Program accession no. PRJNA1031372).^1^

### 2.4 Statistical analysis

The raw data were first trimmed to remove adapter sequences and quality filtered as follows: (1) the raw reads were quality-filtered by fqtrim v0.94; (2) paired-end reads were merged using FLASH v1.2.8; (3) chimeric sequences were filtered using Vsearch v2.3.4. After denoising using the DADA2 plugin within the QIIME2 tool, we obtained feature tables and feature sequences. Then using the SILVA (release 138) classifier, all sequences were annotated to species and the sequences were re-evaluated and those belonging to chimeras, containing extensive homopolymers, and likely originated from organelles, archaea, and eukaryotes were removed. Both alpha diversity and beta diversity were calculated by QIIME2, and the graphs were drawn by R package v3.5.2. Venn diagram, principal component analysis (PCA), correlation analysis and heat maps were plotted using Hiplot,^2^ and the column diagram was drawn using GraphPad Prism v8.0. The Linear discriminant analysis of effect size analysis (LEfSe) was performed using the OmicStudio tools^3^ to determine the observed features that most likely to explain differences between different groups (Segata et al., 2011). In addition, we also used PICRUSt2 v2.1.4 (Douglas et al., 2020) to predict bacterial involvement in metabolic pathways, and used functional annotation of prokaryotic taxa (FAPROTAX) database (Sansupa et al., 2021) to identify bacterial function via the annotation of feature sequence classification.

### 2.5 Isolation and identification of culturable nitrogen-fixing and denitrifying bacteria in *Arthrobotrys musiformis*

Culturable ESB were isolated from axenic *A. musiformis* mycelia as follows. First, two agar plugs (10 mm in diameter) of each axenic *A. musiformis* strain grown on PDA plates were transferred into a 250 mL Erlenmeyer flask containing 100 mL PDB, and these inoculated strains were incubated at 28°C for 5 days, with shaking at 180 rpm. As mentioned above, bacteria were isolated from *A. musiformis* mycelia after confirmation of no contamination. The high-throughput results revealed the rich bacteria species and the presence of N-cycle-associated bacteria in *A. musiformis*. Therefore, we chose three media for isolating bacteria, including Luria-Bertani agar (LB; 10 g tryptone, 5 g yeast extract, 10 g NaCl, 18 g agar, 1,000 mL distilled water) for common bacteria, Ashby medium (Qingdao Hope Bio-Technology, China) for nitrogen-fixing bacteria, and BTB medium (Qingdao Rishui Bio-Technology, China) for aerobic denitrifying bacteria. Second, the collected axenic mycelia were mixed with phosphate buffered saline (PBS; 8 g NaCl, 0.2 g KCl, 1.44 g Na_2_HPO_4_, 0.24 g KH_2_PO_4_, 1,000 mL distilled water), and mechanically broken using Tissue Lyser (Ningbo Xinzhi Biotechnology, China) with 3 × 120 s and 50 Hz parameters. The mixed liquor was filtrated through 5 μm microporous membrane to remove fungal broken cells or conidia, then, 200 μL filtrate was, respectively, spread on 90 mm LB, Ashby and BTB plates and incubated at 30°C. The emerged bacterial colonies were picked and purified, then were maintained in LB Broth (10 g tryptone, 5 g yeast extract, 10 g NaCl, 1,000 mL distilled water). Finally, the obtained pure ESB were deposited in 80% glycerin tube at ultra-low temperature freezer (−80°C) for long-term preservation. Genomic DNA of all isolated ESB was extracted as described by Pakvaz and Soltani (2016). The 16S rDNA was amplified by PCR using the universal primers 515F and 806R as described by Fu et al. (2020) and sequenced by the above LI-COR 4000 L automatic sequencer. Furthermore, the bacterial sequences of each representative species were deposited in GenBank (Supplementary Table S7).

For the identification of all isolated bacterial strains, the obtained sequences were firstly analyzed online using BLASTn in the GenBank database to determine a preliminary classification. Then, Bayesian analysis of the alignment, including our obtained sequences and selected representative sequences of each taxon, was conducted in MrBayes 3.1.2 (Huelsenbeck and Ronquist, 2001) for further identification. *Chlorobium limicola* DSM 245 (belonging to the phylum Chlorobiota) was used as the outgroup.

### 2.6 Investigating the effects of nitrogen-fixing and denitrifying bacteria on the trap formation of *Arthrobotrys musiformis*

Seven common bacteria from ESB capable of either denitrification and nitrogen fixation were selected for co-culture with three *A. musiformis* strains isolated from different habitats, YMF1.07180 (alpine soil), YMF1.01796 (agricultural soil), and YMF1.03736 (sediment). These seven bacterial strains were *Bacillus licheniformis* L3709-1 (BL), *Achromobacter xylosoxidans* L3736-2 (AX), *Stenotrophomonas maltophilia* L7173 (SM), *Sphingomonas paucimobilis* fnBN6HH (SP), *Agrobacterium pusense* fnXS9-3 (AP), *Pseudomonas psychrotolerans* fnBM1Z (PP), *Leclercia adecarboxylata* dnDC7-1 (LA).

Firstly, the seven bacterial strains were cultured in 50 mL of LB medium at 37°C for 24 h, formulating into a 1.67 × 10^7^ CFU bacterial suspension. Three *A. musiformis* strains YMF1.07180, YMF1.01796 and YMF1.03736 were inoculated on PDA plates at 28°C for 5 days, then 200 μL of conidial suspension with a concentration of ~5 × 10^5^ conidia per mL was spread on a 60-mm-diameter water agar (WA, 18 g agar, 1,000 mL distilled water) plate and incubated at 28°C. After 24 h of growth, 100 μL of each bacterial suspension was added to the above WA plate and further incubated for 48 h at 28°C. The negative controls were the equal-volume of sterile water and the experiments were repeated three times. Traps were observed and counted in 10 low-power light microscope fields. For each plate, three random fields of traps were counted within 0.5 cm from the edge of the plate, and the sum of traps in the three fields was recorded.

## 3 Results

### 3.1 Isolation of *Arthrobotrys musiformis*

A total of 97 strains of *A. musiformis* were isolated and purified from diverse habitats, and the detail information of all strains were listed in Supplementary Table S1. All the strains were preserved in 20% glycerol at −80°C for long-term storage. In addition, pure cultures were also deposited in the Herbarium of the Laboratory for Conservation and Utilization of Bio-resources, Yunnan University, Kunming, Yunnan, P.R. China (YMF; formerly Key Laboratory of Industrial Microbiology and Fermentation Technology of Yunnan).

### 3.2 Occurrence of ESB

When we observed the mycelial morphology of several *A. musiformis* strains under fluorescence microscope after fluorescent staining, rapidly moving bodies roughly the size of bacteria were seen in fungal hypha (Sipplementary Movie S1). Then, we randomly selected 20 strains of *A. musiformis* from different habitats for staining and observation, and the bacteria-like bodies (BB) were observed in the hyphal cell and reproductive structure of all selected strains. These BB were clearly seen, meanwhile the larger structures were considered fungal organelles (Supplementary Figure S1). Our results indicated that BB, which we consider to be ESB combined with the rapidly moving bodies roughly the size of bacteria and positive PCR results of total genomic DNA scan, were viable and confirmed viability within hyphae. This finding of ESB in the reproductive structure also demonstrated that ESB can be transmitted vertically to descendants.

Moreover, the total genomic DNA from the fungal cultures were screened for bacterial 16S rRNA, and over 98% of fungal cultures were successfully amplified with single bands (Supplementary Figure S2). These positive PCR products were sequenced, but the sequence analysis showed that most of them contained double peaks (data not shown). Therefore, we subsequently performed high-throughput sequencing to detect ESB in *A. musiformis*.

### 3.3 Sequence-based ESB community diversity within *Arthrobotrys musiformis*

A total of 5,142,740 raw bacterial reads were obtained from the 64 samples of *A. musiformis*. After quality filtering, there were 4,450,231 high-quality sequences used in subsequent analyses, ranging from 59,840 to 78,675 sequences per sample (the average number was 68,244, Supplementary Table S2). All samples had over 99% of average Good’s coverage (an indicator of the sample completeness, Supplementary Table S3). Furthermore, the rarefaction curves showed that the sequencing depth in this study was sufficient to cover the majority of taxa (Supplementary Figure S3).

**Figure 1:**
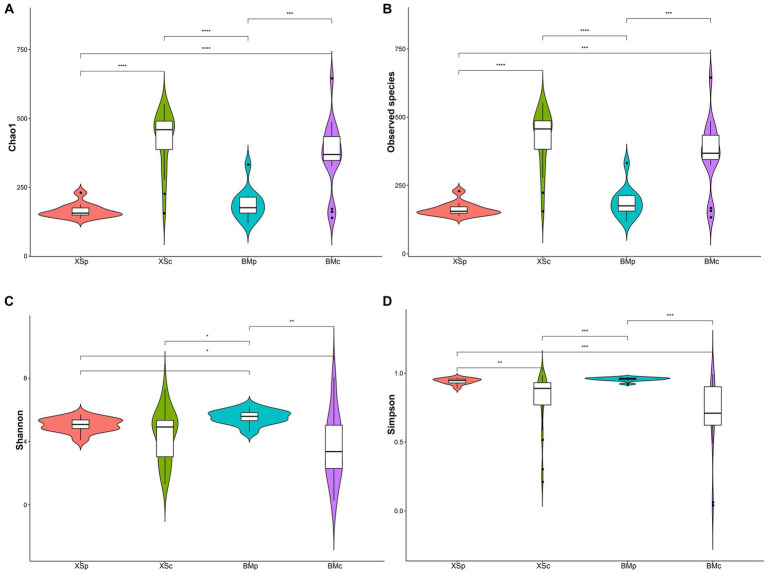
The bacterial community indices for four groups in *A. musiformis*. **(A)** Chao1, **(B)** Observed species, **(C)** Shannon, **(D)** Simpson.

The alpha diversity of the microbial community was assessed by Chao1, Observed species, Shannon and Simpson indices (Soto del Rio et al., 2017). As shown in the Figure 1, these indices indicated that groups XSc and BMc had higher diversity compared with XSp and BMp. This results indicated that *A. musiformis* cultured in CMB has more abundant bacterial communities than in PDB. In contrast, the bacterial species richness and diversity did not differ between the two habitats.

### 3.4 The community structure of ESB in *Arthrobotrys musiformis*

**Figure 2:**
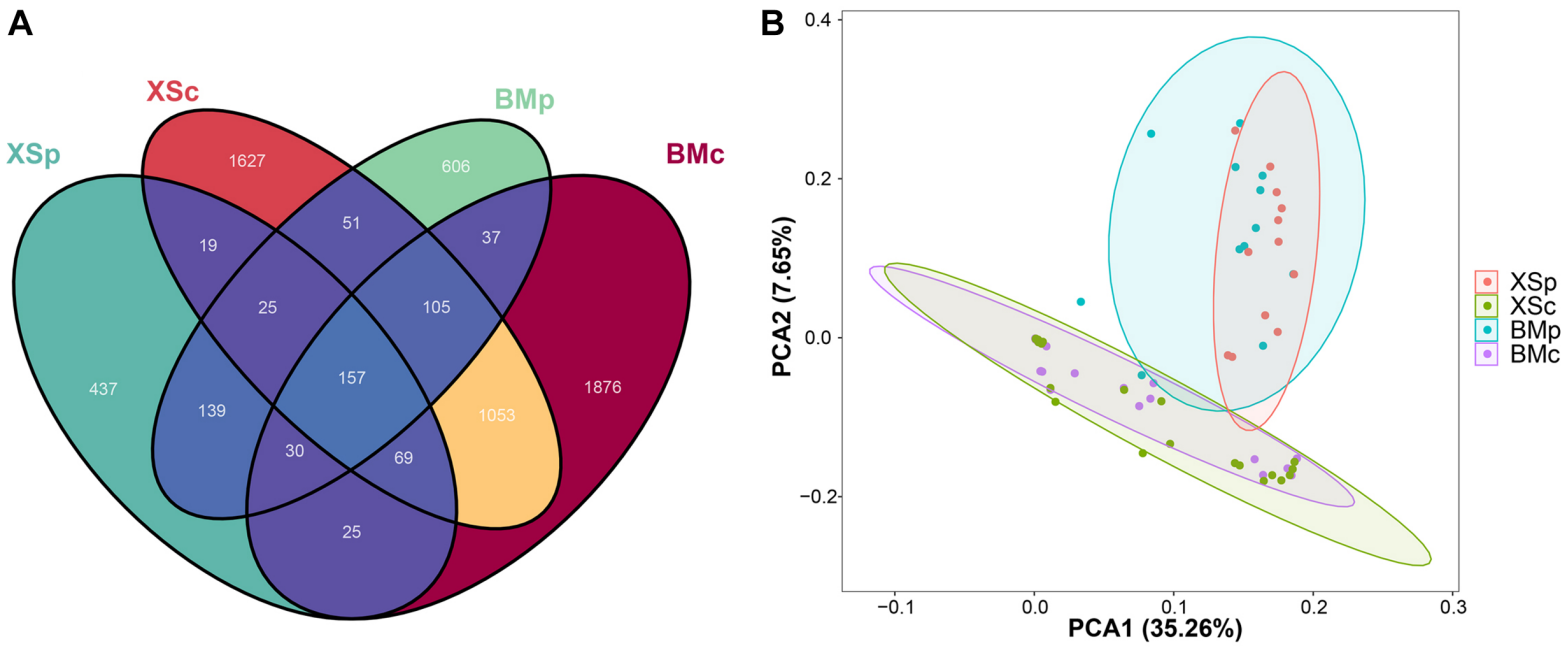
The bioinformatic analysis of bacterial community structure for four groups in *A. musiformis*. Bacterial community structure on ASV level. **(A)** Venn diagram, **(B)** PCA.

Across all samples, the filtered sequences were binned into 5,901 amplicon sequencing variants (ASVs, sequences clustered at a 100% sequence identity), ranging from 118 to 645 per sample (mean = 308; Supplementary Table S2). The group XSc had the highest number of ASVs (3,111), followed by BMc (2,873) and BMp (1,151), and the fewest number was XSp (904). The Venn diagram revealed the overall number of common and unique ASVs among different groups. In Figure 2A, the number of common ASVs was 157, while the numbers of unique ASVs for BMp, XSp, BMc, and XSc were 620, 438, 1,516 and 1,707, respectively. For different habitats, BMp shared 351 ASVs with XSp, accounting for 20.6% of the total ASVs, while BMc shared 1,384 ASVs with XSc, accounting for 27.3% of the total ASVs (Supplementary Figure S4). For different culture media, BMp shared 329 ASVs with BMc, accounting for 7.9% of the total ASVs, while XSp shared 270 ASVs with XSc, accounting for 7.2% of the total ASVs (Supplementary Figure S4). In short, we found that *A. musiformis* host abundant bacterial community (Up to 5,900 ASVs were detected), where more bacterial taxa were detected when cultured in nutrient-poor CMB medium than in rich medium PDB, and different groups cultured in the same medium shared more bacterial taxa than those from the same habitat but cultured in different media.

Further beta diversity analysis was performed to visualize similarity and dissimilarity among sample groups (ASVs; 100% identity) complexity. The PCA plot displayed the bacterial communities among the four groups. Similarly, the structure of bacterial communities was distinct between groups culturing in CMB (BMc and XSc) and PDB (BMp and XSp), and the ESB microbiota of *A. musiformis* was more similar in the same culture medium than from in habitat (Figure 2B). Likewise, a recent study also suggested that different culture conditions play an important role in capturing the diversity of bacterial communities (Li et al., 2023).

### 3.5 Species composition of ESB in *Arthrobotrys musiformis*

According to the annotation of all ASVs of ESB recovered from *A. musiformis*, the ASVs were classified to 35 phyla, which were mainly composed of Firmicutes (32.51%), Proteobacteria (32.32%), Bacteroidota (13.09%), Actinobacteriota (11.49%), Chloroflexi (1.84%), Acidobacteriota (1.23%) and the others 30 rare occurrence phyla (the rest of the phyla with relative abundance less than 1% are set as “others,” 7.08% in total), with unclassified at the phylum level (0.43%) (Supplementary Figure S5; Supplementary Table S4). All ASVs were further assigned to 102 classes, 246 orders, 429 families and 1,010 genera (Supplementary Table S4). Due to the strong representativeness and clear classification level of phylum and genus, we analyzed and compared ESB composition in *A. musiformis* for four groups at the phylum and genus levels hereafter.

**Figure 3:**
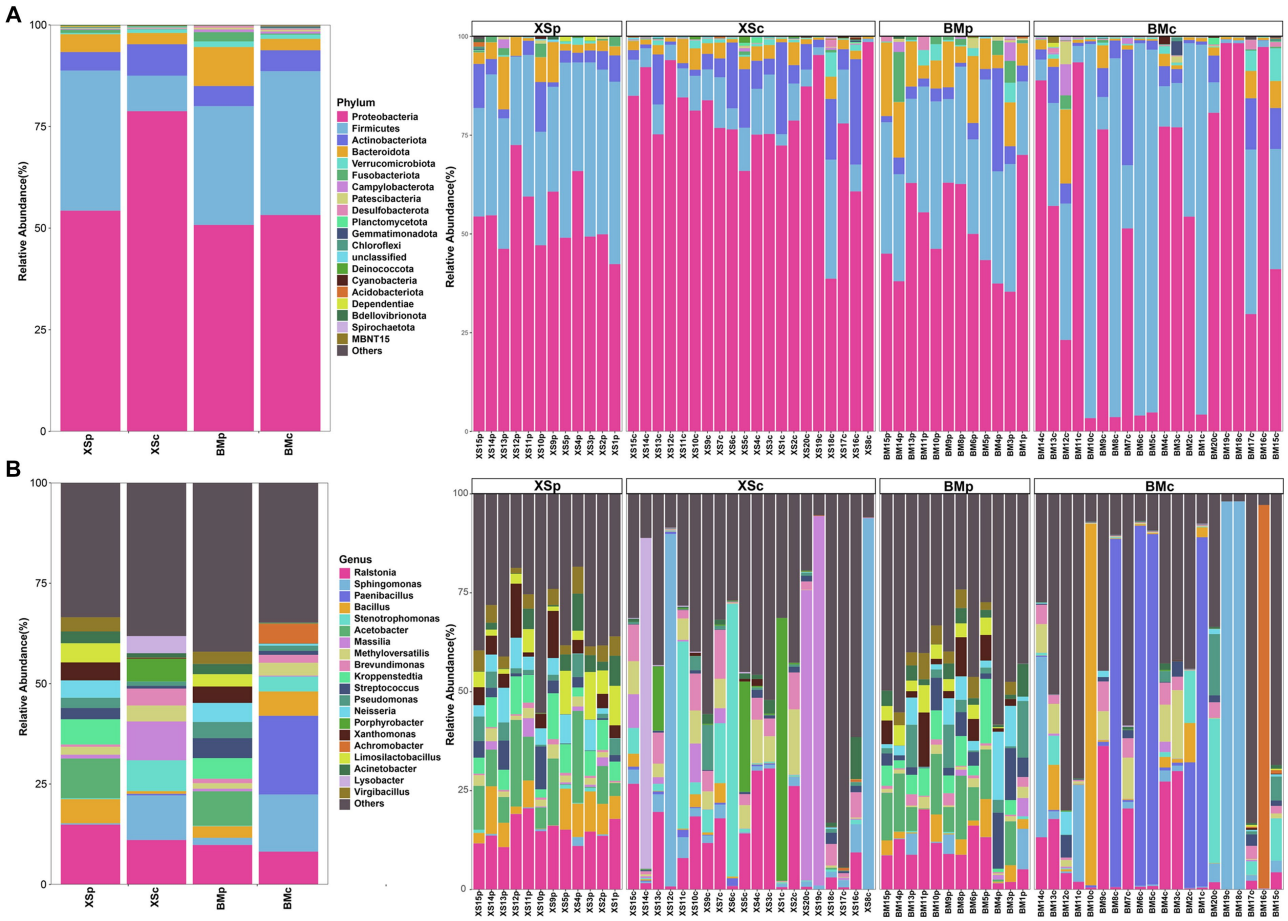
The relative abundance of bacterial community in *A. musiformis* at the phylum (top 20) level **(A)** and the genus (top 20) level **(B)**.

Figure 3 showed the relative abundance of the top 20 bacterial phyla and genera in each sample and group. At the phylum level, Proteobacteria was the predominant phylum in most of the samples except a few samples from BMc, with abundances of 35.39 ~ 70% in BMp, 42.37 ~ 72.53% in XSp, 3.36 ~ 98.33% in BMc and 38.68 ~ 98.59% in XSc, followed by Firmicutes, Actinobacteriota and Bacteroidota (Figure 3A), where they accounted for over 83.31% of the total reads. We also found that the abundance of different phyla is similar and relatively stable between BMp and XSp samples culturing in PDB medium while vary greatly in BMc and XSc samples culturing in CMB medium. For example, Proteobacteria was the most dominant phylum in many *A. musiformis* samples, however, Firmicutes was absolutely dominant in several of the BMc samples (such as BM10c, BM8c and BM6c), which was mainly caused by a surge in the relative abundance of *Paenibacillus*. Previous studies showned that *Paenibacillus* could stimulate the growth of mycelium (Aspray et al., 2013; Oh and Lim, 2018), therefore, we inferred that *Paenibacillus* may help *A. musiformis* grow and develop in nutrient-poor conditions by providing nitrogen or producing stimulatory growth metabolites. At the genus level, the bacterial communities were dominated by *Ralstonia*, *Sphingomonas*, *Paenibacillus*, *Bacillus*, *Stenotrophomonas*, *Acetobacter*, *Massilia*, *Methyloversatilis*, *Kroppenstedtia*, and *Brevundimonas* (Figure 3B). Similarly, the distribution of different genera was more similar between BMp and XSp groups, and the dominant genera of BMc and XSc groups were more variable. In this study, several bacterial taxa, such as *Ralstonia* (Bai et al., 2021) which was previously documented as ESB in other fungi, were not necessarily endosymbiotic with *A. musiformis*, and this suggested that NTF might possibly serve as an alternative niche for bacterial colonization.

### 3.6 Differential bacteria and their correlation network in *Arthrobotrys musiformis* from four groups

**Figure 4:**
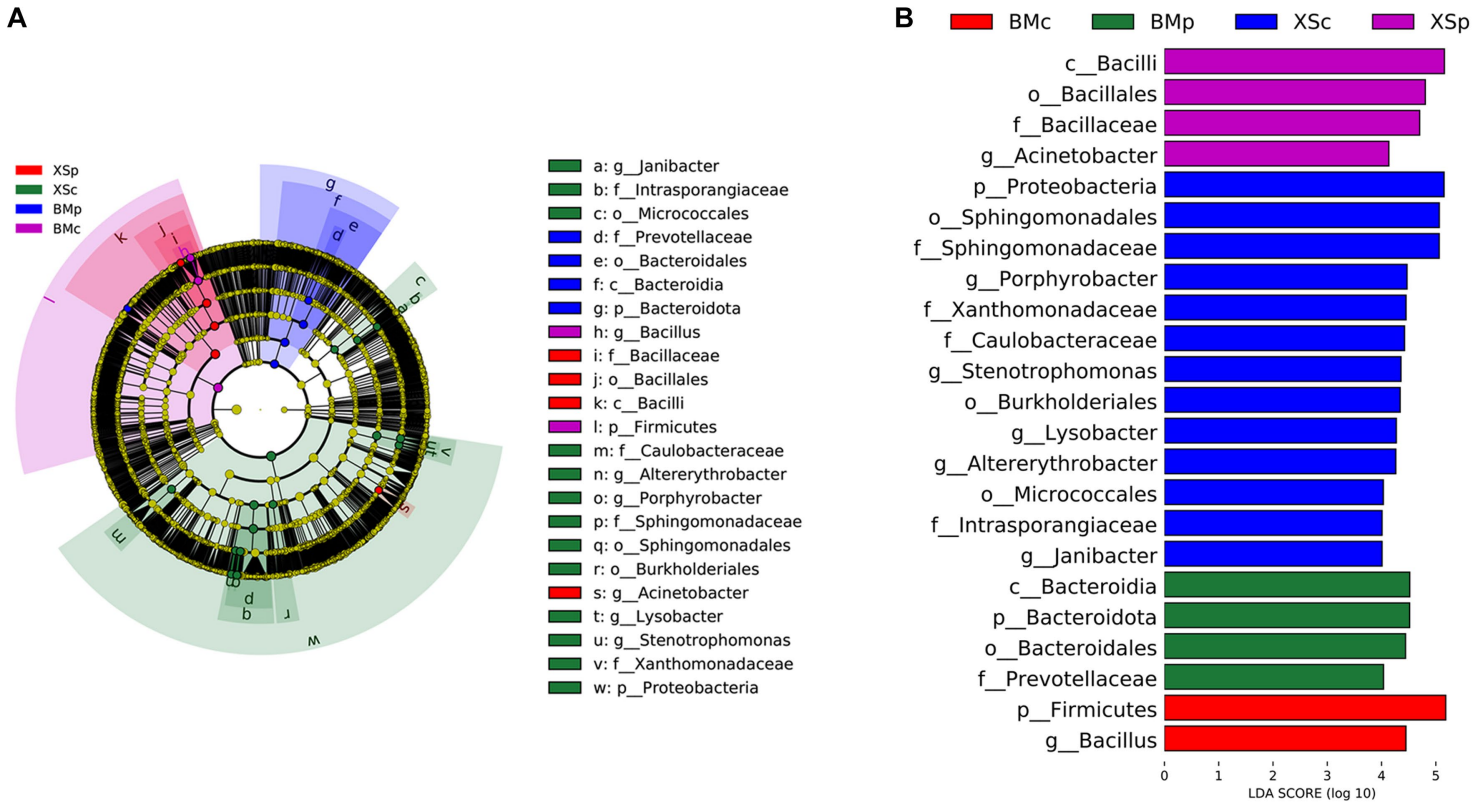
Comparing the bacterial abundance in four groups of *A. musiformis* by LEFSe analysis. **(A)** Five-level cladogram of bacterial communities, **(B)** The extent of the differences in four groups.

LEfse analysis can identify species with significant differences in abundance at all levels among different groups. In this study, LEfSe analysis was performed with a linear discriminant analysis (LDA) threshold of 4.0 and Kruskal-Wallis and Wilcoxon test filtering threshold of 0.05. A total of 23 bacteria (at different classification levels) had statistically significant differences among the four groups, including 3 phyla, 2 classes, 5 orders, 6 families, and 7 genera. The LEfse multi-level species hierarchy tree from phylum to genus was drawn using the difference in bacterial abundance among four groups and is shown in Figure 4A, and the LDA scores of different bacteria in each group are shown in Figure 4B. LEfSe analysis indicated that the discriminating taxonomic units of BMc were Firmicutes and *Bacillus*; those of BMp were Bacteroidales, Bacteroidia, Bacteroidota and Prevotellaceae; those of XSc were Intrasporangiaceae, Sphingomonadaceae, *Altererythrobacter*, Sphingomonadales, *Stenotrophomonas*, Caulobacteraceae, Proteobacteria, Micrococcales, *Porphyrobacter*, Xanthomonadaceae, *Lysobacter*, *Janibacter* and Burkholderiales; those of XSp were Bacilli, *Acinetobacter*, Bacillales and Bacillaceae.

**Figure 5:**
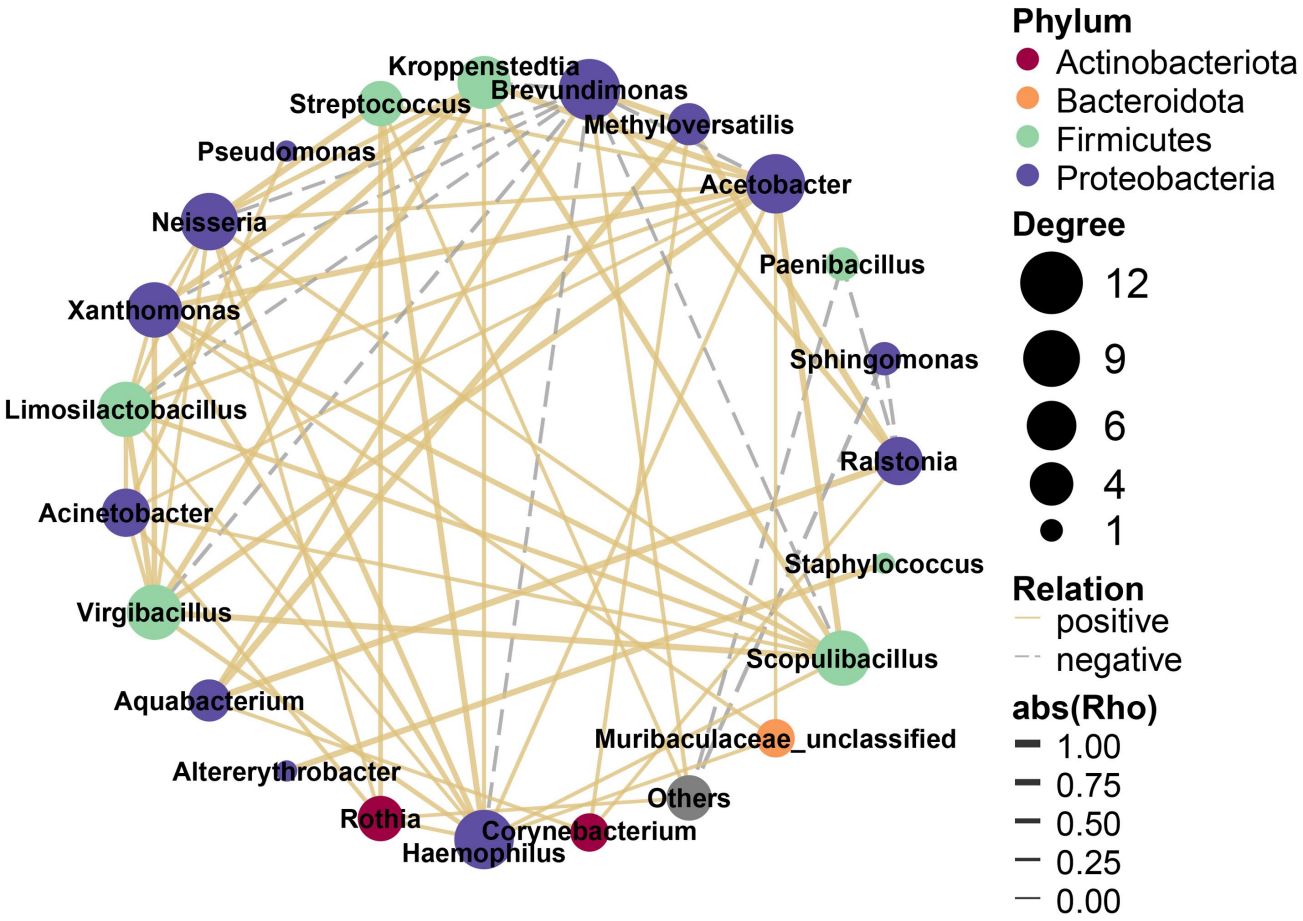
Interaction networks of differential bacteria.

Through the nature of its topological properties, the complex microbial network has been unraveled (Blondel et al., 2008; Paranyushkin, 2012). There were complex correlations among the 22 most abundant bacterial genera, which were assigned to four phyla (Figure 5). Of these, the most highly abundant genera (12 genera) belonged to Proteobacteria, and were significantly positively correlated with other genera except *Brevundimonas* and *Sphingomonas*; five genera belonged to Firmicutes, and four were positively correlated with each other while *Paenibacillus* was negative; two bacterial genera belonged to Actinobacteriota were positively correlated with each other; the only genus of Bacteroidota (Muribaculaceae_unclassified) also correlated with other genera. In general, there were signatures of synergistic and functionally similar connections among the positively correlated genera, while negative correlations may indicate antagonism among these genera.

### 3.7 The core ESB in *Arthrobotrys musiformis*

We compared the bacterial communities at the genus and phylum level among the four *A. musiformis* groups to explain the selectivity properties of different habitats and culture media. Identifying the core ESB is important to capture the key microbes that are closely associated with a host population, as this process can potentially help to further elucidate their spatial distribution, temporal stability, ecological influence and even impact on their host’s functions and fitness. Although the bacterial communities in *A. musiformis* displayed heterogeneity in bacterial composition among different strains and groups, 157 shared ASVs were found in four groups. The 157 ASVs were referred as core bacterial taxa in *A. musiformis* hereafter. After annotation, the core bacterial taxa distributed in 10 phylum and 101 genera (Supplementary Table S5). Of these, most ASVs belonged to Proteobacteria (68 ASVs), which made up 59.28% of the mean relative abundance in four groups, followed by Firmicutes (45 ASVs, 26.95%), Actinobacteria (11 ASVs, 5.60%), Bacteroidota (24 ASVs, 4.88%), Bdellovibrionota (2 ASVs, 0.90%), Desulfobacterota (2 ASVs, 0.24%), Verrucomicrobiota (2 ASVs, 0.05%), Campylobacterota (1 ASVs, 0.37%), Deferribacterota (1 ASVs, 0.01%), and Fusobacteriota (1 ASVs, 0.87%).

Of the 101 genera, 10 genera were highly abundant (belonging to top 30) genera in *A. musiformis*, namely *Acinetobacter*, *Massilia*, *Sphingomonas*, *Streptococcus*, *Brevundimonas*, *Neisseria*, *Pseudomonas*, *Methyloversatilis*, *Ralstonia*, and *Xanthomonas*, contributing 30.87 ~ 43.1% of the bacterial microbiome in the four groups (Table 1). *Sphingomonas* (made up 15.74% of the mean relative abundance in four groups), *Ralstonia* (9.66%), and *Methyloversatilis* (4.70%) were the three most abundant genera within the core bacterial taxa. We also found greater differences in the abundance of many core genera under different media compared to different habits. For instance, the relative abundance of *Sphingomonas* was significantly higher in CMB medium (*p* = 0.021), whereas *Neisseria* (*p* = 0.005) and *Xanthomonas* (*p* = 0.002) were more abundant in PDB medium.

**Table 1:** The relative abundance of the top 10 core genera in four groups.

Genus	Relative abundance (%)				Result of ANOVA test			
					Medium		Habitat	
	BMc	BMp	XSc	XSp	*F*	*p*	*F*	*p*
*Ralstonia*	8.16	9.83	11.06	14.90	0.89	0.445	3.623	0.197
*Sphingomonas*	14.24	1.78	11.12	0.34	45.74	0.021	0.077	0.808
*Massilia*	0.23	0.62	9.65	0.95	0.777	0.471	1.253	0.379
*Methyloversatilis*	3.20	1.37	3.98	1.97	15.227	0.060	0.258	0.662
*Brevundimonas*	1.97	1.11	4.22	0.57	3.799	0.191	0.208	0.693
*Streptococcus*	1.00	4.97	0.67	2.82	7.916	0.107	0.302	0.638
*Pseudomonas*	1.28	4.01	1.04	2.53	7.922	0.106	0.306	0.636
*Neisseria*	0.50	4.76	0.04	4.34	188.849	0.005	0.021	0.898
*Xanthomonas*	0.05	4.09	0.17	4.47	438.008	0.002	0.007	0.940
*Acinetobacter*	0.24	2.55	1.15	2.94	17.150	0.054	0.198	0.700
Total	30.87	35.09	43.10	35.83				

### 3.8 Microbial nitrogen metabolism

Notably, the bacterial genera *Bacillus* and *Pseudomonas*, in which many species are capable of nitrogen fixation and denitrification (Hino and Wilson, 1958; Carlson and Ingraham, 1983; Verbaendert et al., 2011), were abundant in *A. musiformis*. Therefore, we used PICRUSt2 and FAPROTAX to predict ESB function and mainly focused on nitrogen metabolism process.

**Figure 6:**
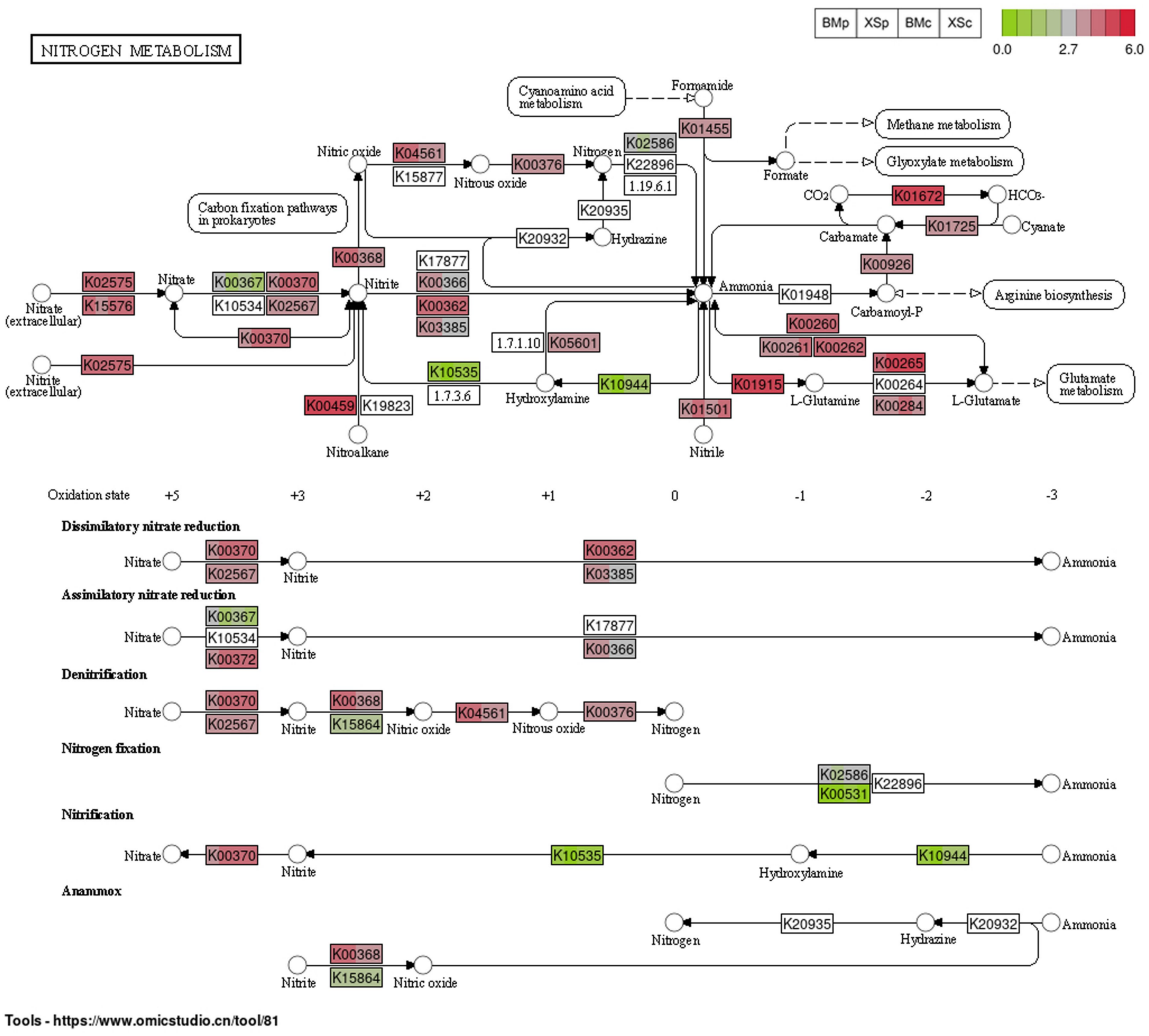
The abundance of the predicted functional genes of the nitrogen metabolism pathway (map00910) for the four groups in *A. musiformis*, predicting by PICRUSt2. Each rectangle labeled with the functional protein number of KEGG is divided into four color blocks, one representing a group (in the order BMp, XSp, BMc, and XSc), allowing comparison of the abundance of a gene in different groups. Blank rectangles indicated the absence of the gene.

Based on 16S rRNA gene-based microbial compositions and abundance information, we used PICRUSt to make inferences the nitrogen metabolism pathways. The nitrogen metabolism map was constructed based on the abundance of the functional genes of the nitrogen metabolism pathway (map00910) for the four groups and was shown in Figure 6. Microbial nitrogen transformations are often presented as a cycle consisting of six distinct processes, including nitrogen fixation, nitrification, denitrification, anammox, assimilation and dissimilation (Kuypers et al., 2018). Our study showed that ESB in *A. musiformis* have the potential to function in five modules of the nitrogen metabolism, except for anammox. The functional genes related to other modules nitrification, denitrification, assimilation and dissimilation had high abundance within four groups (BMp, XSp, BMc, and XSc) of *A. musiformis*, such as K01915/K00265 for glutamate synthesis and K02575 for nitrate/nitrite transport system, but the genes K10535/K10944 for nitrification and K00531 for nitrogen fixation had low abundance in four groups. It was worth noting that the genes K03385 for dissimilatory nitrite reduction, K00366 for assimilatory nitrite reduction, and K00376 for nitrous oxide reduction has higher abundance in nutrient-rich culture media (BMp and XSp). Briefly, the nitrogen cycle is a process in which dinitrogen is converted to ammonia, ammonia is oxidized to nitrate, and nitrate is reduced to ammonia or dinitrogen. Although the absence of genes k20935 and k20932 prevented anommox, the other five modules of the nitrogen metabolism in ESB essentially completed the N-cycle process.

According to FAPROTAX analysis, 2,291 ASVs, which cover 38.82% of all sequences, were assigned to 72 functional groups, including 15 N-cycle groups (Supplementary Table S6). As shown in Supplementary Figure S6, the predicted nitrate reduction was the most abundant function in *A. musiformis*, which has higher abundance in nutrient-rich PDB medium (BMp and XSp) compared with nutrient-poor CMB medium (BMc and XSc), followed by nitrate respiration, nitrogen respiration and ureolysis.

### 3.9 Cultured and characterized nitrogen-fixing and denitrifying bacteria from *Arthrobotrys musiformis*

**Figure 7:**
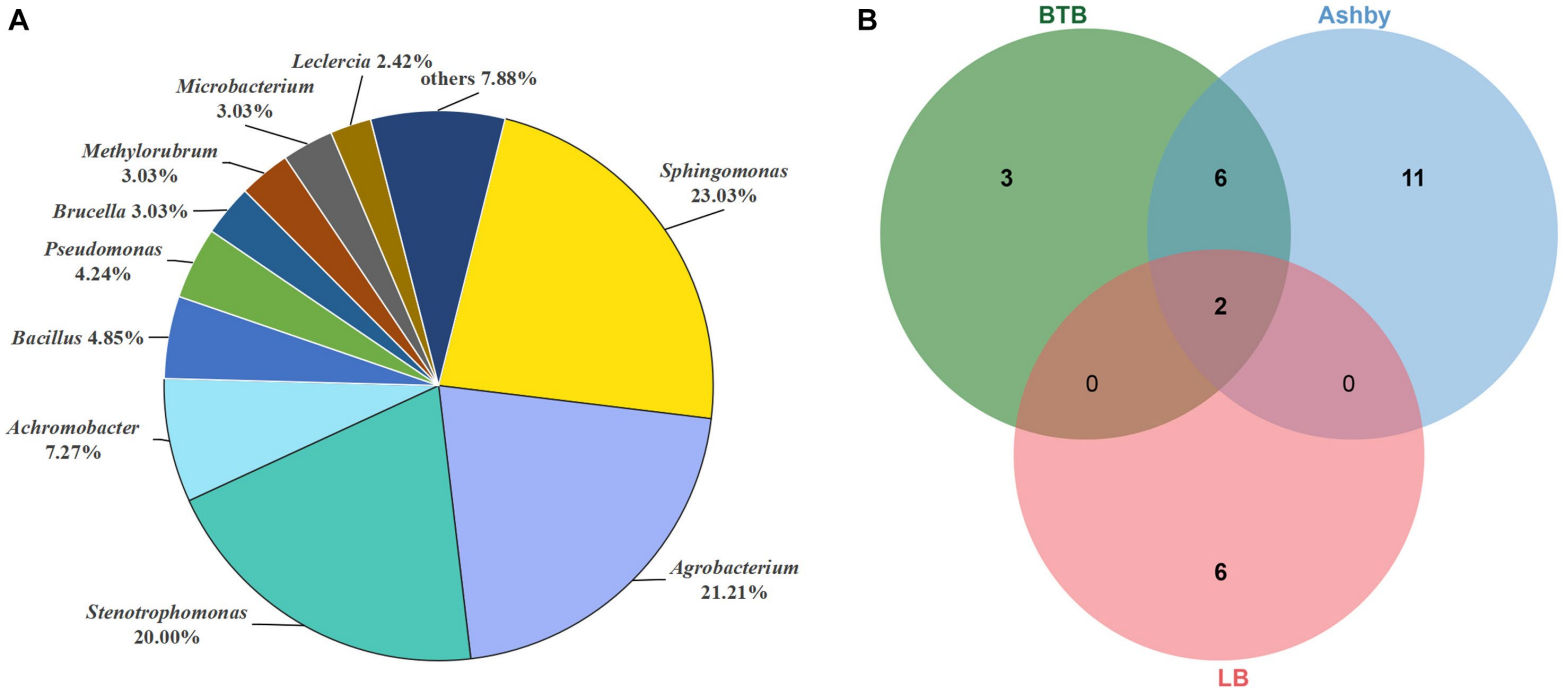
Distribution of culturable ESB in *A. musiformis*. **(A)** The total distribution of bacterial genera in *A. musiformis*, **(B)** Venn diagram of bacterial species isolated from the three media BTB, Ashby and LB.

We isolated a total of 165 bacterial strains from broken cells of *A. musiformis* used three media: 48 strains from LB, 55 strains from Ashby, 62 strains from BTB. The 16S rRNA gene sequence was obtained from each strain and BLASTn search was used to preliminarily determine its classification in genus level. Our analyses assigned these 165 strains to 20 genera belonging to four phyla (Pseudomonadota 89.7%, Bacteroidota 1.82%, Actinomycetota 3.03%, and Bacillota 5.45%). A phylogenetic tree was constructed to further identify these ESB at species level (Supplementary Figure S7). These 165 strains were identified as belonging to 28 species and the detailed information was given in Supplementary Table S7. The dominant genera of culturable ESB were *Sphingomonas* (38 strains, 23.03%), *Agrobacterium* (35 strains, 21.21%) and *Stenotrophomonas* (33 strains, 20%), with *Ag. pusense* (34 strains), Sp. *paucimobilis* (32 strains) and *St. maltophilia* (32 strains) as the dominant culturable species in *A. musiformis* (Figure 7A). Among these bacteria, most were successfully sub-cultured and preserved. However, a few lost viability during subculturing, consistent with previous observations that many obligate ESB are unculturable (Salvioli et al., 2008).

When comparing the number of bacterial species isolated from the three media (Figure 7B), it was found that the highest number of species were isolated from Ashby (19 species), followed by BTB (11 species), and the fewest number of species were isolated on LB (8 species). Furthermore, BTB and Ashby shared many species, accounting for 38.09% of its total species, and the result suggested that these bacterial species are capable of both denitrification and nitrogen fixation. However, culturable ESB were still limited, and the different media had significant influence on the number and species of culturable bacteria.

In short, we isolated 165 ESB strains based on three media, finally, these strains were classified into 28 species, 20 genera, 15 families, 12 orders, 7 classes, and four phyla. The culturable results reconfirmed that *A. musiformis* has a rich ESB diversity and that some culturable bacteria are involved in nitrogen-cycling processes.

### 3.10 Effects of nitrogen-fixing and denitrifying bacteria on the trap formation of *Arthrobotrys musiformis*

**Figure 8:**
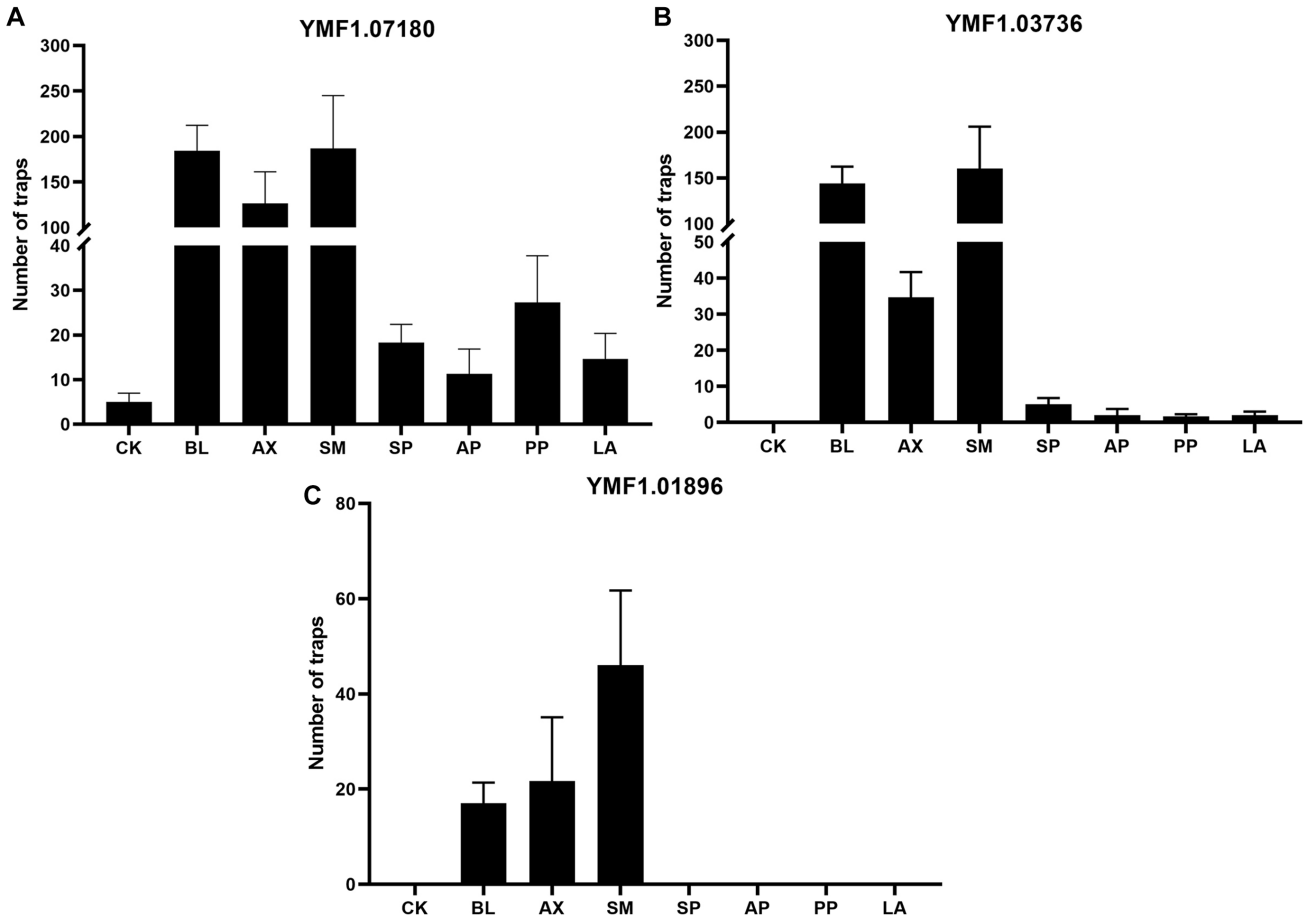
Effects of the eight culturable ESB isolates on the traps of three *A. musiformis* strains. Sterile water treatment was used as a control (CK). **(A)** The effect of the seven bacteria on the traps information of *A. musiformis* YMF1.07180, **(B)** The effect of the seven bacteria on the traps information of *A. musiformis* YMF1.03736, **(C)** The effect of the seven bacteria on the traps information of *A. musiformis* YMF1.01896.

An important indicator of the switch from a saprophytic to a predatory lifestyle is the formation of traps by NTF (Yang et al., 2011). Many bacteria have been reported to be capable of inducing trap formation in NTF, such as cow dung bacteria (Wang et al., 2014) and soil bacteria (Li et al., 2011). We studied the effects of seven culturable and dominant bacteria capable of both denitrification and nitrogen fixation on trap formation in three strains of *A. musiformis*. The sterile water treatment was used as a control (CK), and the results were shown in Figure 8.

We found that *A. musiformis* YMF1.07180 spontaneously produce traps with sterile water treatment (CK) after 3 days of culture, while the other two strains YMF1.03736 and YMF1.01896 did not. All seven bacterial species increased the number of traps in YMF1.07180, and the enhancement effect varied among different bacteria. The three bacteria BL, AX and SM had a strong trap-enhancing effect on YMF1.07180, while the other four bacteria were weaker. For instance, following co-culture with SM, BL, and AX, the number of traps in YMF1.07180, respectively, increases to 187, 184, and 126 (mean, CK: 5), but co-culture with PP, SP, AP, and LA only increase to 27, 18, 11, and 14, respectively, (Figure 8A). In addition to increase the trap formation (Supplementary Figure S8), we also observed the increase in mycelium density after co-culturing with bacteria, and the bacterial treatment group produced more conidia, whereas CK produced none or very few. The CK of YMF1.03736 was not observed to produce traps, but all eight bacteria species could induce it to produce traps. Likewise, the three bacteria BL, AX, and SM were the most efficient inducers, but the others only induced fewer traps. Following co-culture with SP, AP, PP, and LA, the numbers of traps in YMF1.03736 were only 5, 2, 2, and 2 (Figure 8B). For the *A. musiformis* strain YMF1.01896, the trap formation was not observed in the CK, SP, AP, PP, and LA treatment groups, and the numbers of traps induced by the three species BL, AX, and SM were significantly lower than YMF1.07180 and YMF1.03736 (Figure 8C).

These results indicated that the ability of *A. musiformis* to produce traps varies from strain to strain. A recent study reported that some strains of *A. oligospora* have evolved to become intrinsically more robust and efficient in sensing nematodes, executing the trapping developmental program, and consuming prey (Yang et al., 2020). What’s more, all seven bacterial strains enhanced or induced the trap formation of *A. musiformis*. The different bacterial species had different effects on the induction of the same *A. musiformis* strain, and the same bacterial species also had different effects on different *A. musiformis* strains. Overall, the three bacteria BL, AX, and SM were the most efficient inducers.

## 4 Discussion

### 4.1 Bacterial communities in *Arthrobotrys musiformis* displayed high diversity and heterogeneity

Compared with the relatively high bacterial diversity observed in terrestrial plants and animals [e.g., earthworm gut harbor >10^4^ bacterial taxa (Sapkota et al., 2020; Zhu et al., 2022)], some case-studies have reported less ESB diversity in fungi, such as in yeast (Indu et al., 2021) and arbuscular mycorrhizal fungi (Lastovetsky et al., 2018). Here, a total of 5,901 ASVs, which were assigned to 35 phyla, 102 classes, 246 orders, 429 families, and 1,010 genera, were observed from 64 samples of *A. musiformis*, and 160 culturable ESB strains were obtained, which were assigned to 28 species, 20 genera, 15 families, 12 orders, 7 classes, and four phyla. Notably, the 20 culturable genera were also detected by high-throughput sequencing, confirming the accuracy of the high-throughput method.

In this study, our results indicated that more bacterial species could be detected when *A. musiformis* strains cultured in nutrient-poor medium. Therefore, we hypothesized that ESB plays an important role in helping the host survive harsh conditions. The PCoA analysis suggested that the culture media could influence the composition of the ESB assemblages, yet fungal strains from the same habitat but grown in different media displayed high dissimilarity, indicating that the strain sources were not a critical determinant for their ESB microbiome structures. The bacterial research on yeast had found that the bacterial diversity did not show any dependence on environmental conditions from which the yeast hosts were isolated, instead, the diversity was specific to the yeast host itself (Indu et al., 2021). Other studies have drawn similar conclusions (Benucci et al., 2019; Bai et al., 2021; Deng et al., 2023). The structure of ESB communities within ectomycorrhizal and saprotrophic mushrooms showed significant differences across fungal phylogenetic groups and to a lesser extent across fungal guilds, with *Rhizobium* and members of the Burkholderia-Caballeronia-Paraburkholderia complex dominated in fruitbodies (Pent et al., 2020). Meanwhile, the most abundant bacterial genera in *Morchella ascocarps* were *Pedobacter*, *Flavobacterium*, and *Pseudomonas* (Benucci et al., 2019). Specifically, the ESB communities in the NTF *A. musiformis* were dominated by *Ralstonia*, *Sphingomonas*, *Paenibacillus*, *Bacillus*, and *Acetobacter* in this study. In addition, the relative abundance of bacterial taxa at the genus level showed that bacterial taxa varied greatly among different samples. Collectively, these results indicated that bacterial communities of *A. musiformis* displayed high diversity and heterogeneity.

### 4.2 *Arthrobotrys musiformis* recruits a core set of constitutive bacterial taxa with nitrogen metabolism capability

Despite high diversity and heterogeneity, bacterial communities in *A. musiformis* from different habitats and culture media shared a core set of taxa. In this study, 157 bacterial ASVs were widely shared and they distributed in 10 phyla and 101 genera. Among them, 10 genera were highly abundant in *A. musiformis*, namely *Ralstonia*, *Sphingomonas*, *Massilia*, *Methyloversatilis*, *Brevundimonas*, *Streptococcus*, *Pseudomonas*, *Neisseria*, *Xanthomonas*, and *Acinetobacter* (Table 1).

Although the fact that *Ralstonia* is a genus that is known to harbor soil-borne bacterial pathogens, we detected a high abundance (8.16 ~ 14.9% relative abundance in four groups) of *Ralstonia* in all *A. musiformis* samples. The recent study by Bai et al. (2021) also found the high abundance of *Ralstonia* in the core microbiome of the macro-fungus bolete. Further studies will be needed to decipher the impact of these ‘enriched’ microbes on its fungal host. Both *Sphingomonas* and *Methyloversatilis* were more abundant in these *A. musiformis* samples cultured in CMB medium. In contrast, the relative abundances of *Streptococcus*, *Pseudomonas*, *Neisseria*, *Xanthomonas*, and *Acinetobacter* were higher when cultured in PDB medium than on CMB medium. *Methyloversatilis* are defined as methylotrophic bacteria that can participate in the denitrification process (Ontiveros-Valencia et al., 2013). *Sphingomonas* is Gram-negative bacterial genus, and is ubiquitous in the environment such as aquatic system, endophytes, and soil. In 2010, *Sphingomonas* as ESB was detected in endophytes *Phoma glomerata* (Hoffman and Arnold, 2010). Surprisingly, *Streptococcus* and *Pseudomonas* as human and animal (sometimes plant) pathogens were also discovered in healthy fruiting bodies of *Morchella* and *Heterobasidion* (Benucci et al., 2019; Ren et al., 2022). It was worth noting that *Massilia* and *Brevundimonas* are among the most frequently detected bacteria taxa and were also found in isolates from all four fungal phyla examined in a recent study (Robinson et al., 2021). Several species of *Massilia* were able to reduce nitrate (Zhang et al., 2006), suggesting an important role of the genus in the nitrogen cycle. A study reported that *Brevundimonas*, which can proceed nitrification and denitrification, promote the growth of *Chlorella ellipsoidea* and form a symbiotic relationship (Park et al., 2008). *Neisseria* is related Gram-negative bacteria and a facultative commensal that can also infect humans. Genome analysis indicated that *Neisseria* species can mobilize nitrogen from ammonia to amino acids (Schoen et al., 2014). *Acinetobacter*, a common soil bacterium, could colonize plants by their roots and multiply extensively in the pathogen-infected tissues (Kay et al., 2002), and some species exhibited efficient heterotrophic nitrification and aerobic denitrification ability (Yang et al., 2015). Several previous studies on plant endophytic bacteria had identified *Acinetobacter* as one of the dominant genera (Izumi, 2011), such as in tree peony roots (Yang et al., 2017). Moreover, *Acinetobacter* had been detected in several fungal species (Shaffer et al., 2016; Benucci et al., 2019; Robinson et al., 2021). Collectively, these findings suggest that *A. musiformis* recruits a suite of constitutive bacterial taxa with nitrogen metabolism capability.

### 4.3 Functional prediction of ESB reveals potential nitrogen cycle

Studies examining ESB using high-throughput techniques have rapidly increased in recent years, but functional analyses of ESB are still rare. The entire dataset assigned to 5,901 ASVs revealed an extraordinary diversity of bacterial communities in *A. musiformis*. Particularly concerning the host that bears the cost of harboring ESB, breakthroughs were required to establish the functional contribution of these bacteria in complex symbioses. Therefore, PICRUSt2 were used to predict bacterial function in this study. Our analyses revealed limited differences in functional groups between fungal strains from different habitats (data not shown), but these groups growing in different media showed significant functional differences (Supplementary Figure S9). Some taxa may influence microbial function by selectively regulating community diversity and composition (Banerjee et al., 2018; Kurm et al., 2019). For the groups BMc and XSc, these enriched functional genes may play important role in helping the host cope in the absence of an important nutrient.

Bacterial diversity and composition are ecologically important for fundamental ecosystem processes such as microbial nitrogen metabolism and nutrient cycling (Crowther et al., 2019; Delgado-Baquerizo et al., 2020). Ruiz-Herrera et al. (2015) firstly observed the existence of an endosymbiotic N_2_-fixing bacterium in the maize pathogenic fungus *Ustilago maydis* in 2015. Later, Koch et al. (2021) also found that the basidiomycete *Guyanagaster necrorhizus* harbors active N_2_-fixing Enterobacteriaceae species. More recently, the endosymbiont *Bacillus tequilensis* was reported in *Kluyveromyces marxianus*, and it provides assimilable nitrogen to host under nitrogen starvation conditions. In fact, many fungal species grew in nitrogen-free media at a rate similar to that observed in media containing ammonium nitrate, suggesting that this may be the role of its endosymbiont. The high-throughput sequencing results showed that *A. musiformis* contains a high abundance of bacterial taxa involved in nitrogen metabolism, such as *Bacillus* and *Pseudomonas* (Hino and Wilson, 1958; Carlson and Ingraham, 1983; Verbaendert et al., 2011), which are capable of nitrogen fixation and denitrification. Therefore, we investigated the potential nitrogen metabolism processes for these ESB. The predicted results showed that ESB in *A. musiformis* have the potential to function as five modules of the nitrogen metabolism pathway, including nitrogen fixation, nitrification, denitrification, assimilation, and dissimilation (Figure 6). FAPROTAX analysis also predicted 15 N-cycle groups for the ESB in *A. musiformis* (Supplementary Figure S6; Supplementary Table S6). Besides, some denitrifying and nitrogen-fixing strains were isolated based on culture-dependent method, confirming the ability of some ESB of *A. musiformis* to participate in nitrogen metabolism. The gut microbiota (e.g., in earthworm) has always been a research hotspot. The anoxic gut environment in earthworm provided an ideal and stable space for the survival and colonization of anaerobic or facultative anaerobic bacteria, that favor anammox and denitrification processes (Li et al., 2021). Likewise, fungi also provide a special environment for its ESB. Together, our results reveal that ESB in *A. musiformis* are capable of broad and diverse functional potentials, which still required further experimental validation. It is also the first to unveil that ESB has potential to involved in more nitrogen metabolism pathways. Moreover, we realized that the current evidence from this and previous studies fall short in addressing the function of ESB in NTF.

### 4.4 The role and effect of bacteria on NTF

Logically, the extensive occurrence of bacteria in *A. musiformis* revealed here implies that their interactive relationships should be seen as either mutually beneficial or unilaterally beneficial but harmless to the other. Both scenarios favor long-term and stable co-existence rather than the exclusion of one or the other. ESB can affect spore shape (Araldi-Brondolo et al., 2017), spore production (Mondo et al., 2017) and also make the mycelium of fungal hosts thicker and more lush (Araldi-Brondolo et al., 2017), in addition to playing an important role in the metabolism and resilience of fungal hosts. So far, several studies have suggested that bacteria play an important role in the transition of NTF to a parasitic lifestyle, although this transition was thought to be the result of some competition for nutrients between fungi and bacteria (Persmark and Nordbring-Hertz, 1997). Early on, Cayrol et al. (1984) found the gut bacteria of nematodes can induce or increase trap formation of *A. oviformis*. Then, Rucker and Zachariah (1987) investigated the effects of eight bacteria, which were *Bacillus subtilis*, *Micrococcus* sp., *Staphylococcus aureus*, *Pseudomonas aeruginosa*, *Serratia marcescens*, *Azotobacter* sp., *Proteus vulgaris*, and *Escherichia coli* respectively, on *D. brochopaga*, and their results showed that different bacterial species evoke very different numbers of traps in various *D. brochopaga* strains. Furthermore, they also found different bacteria have important effect on hyphal growth of NTF. These bacteria, *Chryseobacterium* sp. isolated from soil and *Stenotrophomonas maltophilia* isolated from cow dung, induced trap formation in *A. oligospora* (Li et al., 2011; Wang et al., 2014). However, the effects of ESB in NTF had not yet been investigated, until now.

In this study, the effects of the seven dominant culturable ESB capable of both denitrification and nitrogen fixation, *B. licheniformis*, *Br. pseudogrignonensis*, *Ac. xylosoxidans*, *St. maltophilia*, Sp. *paucimobilis*, *Ag. pusense*, *Ps. psychrotolerans*, *L. adecarboxylata*, co-cultured with *A. musiformis* were investigated. The results showed that all seven bacteria induce or increase trap formation (Figure 8), and ESB had a stimulatory effect on fungal growth (Supplementary Figure S8). As mentioned above, our results confirm the effects of *Stenotrophomonas* and *Pseudomonas* species on trap formation of NTF. Furthermore, different bacteria have different effects on different strains of *A. musiformis*, and this result was consistent with precious studies. For example, Rucker and Zachariah (1987) observed that *Pseudomonas* caused a 1900% increase in trap numbers in one strain of *D. brochopaga*, but only a 4.5% increase in the other strain, nevertheless, they also found that a particular bacterial species could promote trap formation in some fungal isolates while depressing it in others. Noteworthily, many reported inducers of trap formation were nitrogenous substances, such as nematode extract, amino acids, and small peptides. The bacterium *St. maltophilia* was reported to induce trap formation in NTF by secreted urea, and ammonia, a metabolite of urea, had the same effect (Wang et al., 2014). The seven bacteria had the ability to fix nitrogen and denitrify. Therefore, we speculate that nitrogen metabolites produced by ESB may play an important role in NTF predation of nematodes.

In addition, Fu et al. (2020) investigated the diversity and function of endo-bacteria in *Bursaphelenchus xylophilus* by high-throughput sequencing of 16S rDNA and isolated culturable endo-bacteria, and bacteria belonging to six genera, which, respectively, were *Stenotrophomonas*, *Pseudomonas*, *Kocuria*, *Microbacterium*, *Rhizobium*, and *Leifsonia*, were obtained. They found that *P. fluorescens* significantly increased the egg production of pine wood nematode, and that both *P. fluorescens* and *St. maltophilia* enhanced the mobility of pine wood nematodes under oxidative stress. *Stenotrophomonas* and *Pseudomonas* species are widely distributed in a variety of habitats. On the one hand, these bacteria as ESB of NTF can promote mycelial growth and enhance pathogenicity of host fungi. On the other hand, they can enhance egg production and resistance of nematodes as its endo-bacteria. We tried to cure bacteria in *A. musiformis* using a range of antibiotics, but ultimately found that there were too many bacteria to cure. Overall, these results suggested that bacteria, NTF and nematodes have a quite complex relationship.

## 5 Conclusion

Here, we discovered that bacterial communities in *A. musiformis* display high diversity and heterogeneity and that growth media play an important role in capturing the diversity of bacterial communities by using high-throughput sequencing and culture-dependent approaches. To our knowledge, this is the first study to reveal the existence of large bacterial communities in the NTF. The species composition and core taxa of ESB unveiled that *A. musiformis* recruits a suite of constitutive bacterial taxa with nitrogen metabolism capability. PICRUSt2 and FAPROTAX analysis consistently predicted a broad and diverse functional repertoire of the whole bacterial communities in *A. musiformis* and revealed that ESB have the potential to function in five modules of nitrogen metabolism. We also found that seven dominant ESB can induce or increase trap formation of *A. musiformis*. Our results provide novel insights on the relationship and interaction between ESB and NTF and inspire further work into the function and the relevance of individual ESB, including how to enter and exit NTF cells.

## Data availability statement

Publicly available datasets were analyzed in this study. This data can be found at: [https://www.ncbi.nlm.nih.gov/search/all/?term=PRJNA1031372].

## Author contributions

HZ: Formal analysis, Writing – original draft. TC: Formal analysis, Investigation, Writing – review & editing. WL: Formal analysis, Investigation, Writing – review & editing. JH: Formal analysis, Investigation, Writing – review & editing. JX: Funding acquisition, Writing – review & editing. ZY: Conceptualization, Funding acquisition, Writing – review & editing.

## Footnotes

^1^ https://www.ncbi.nlm.nih.gov/bioproject/?term=PRJNA1031372

^2^ https://hiplot-academic.com

^3^ https://www.omicstudio.cn/tool
