# Supplementary material for: Endosymbiotic bacteria within the nematode-trapping fungus Arthrobotrys musiformis and their potential roles in nitrogen cycling
Source: Front Microbiol. 2024 Jan 29;15:1349447. doi: 10.3389/fmicb.2024.1349447 (PMC10860758; doi:10.3389/fmicb.2024.1349447)

**Supplementary figures**

**Figure S1.** ESB (arrow) in hyphae (A and B) and conidia (C and D) of *A. musiformis* were observed under fluorescence microscope after stained with SYTO9.

**
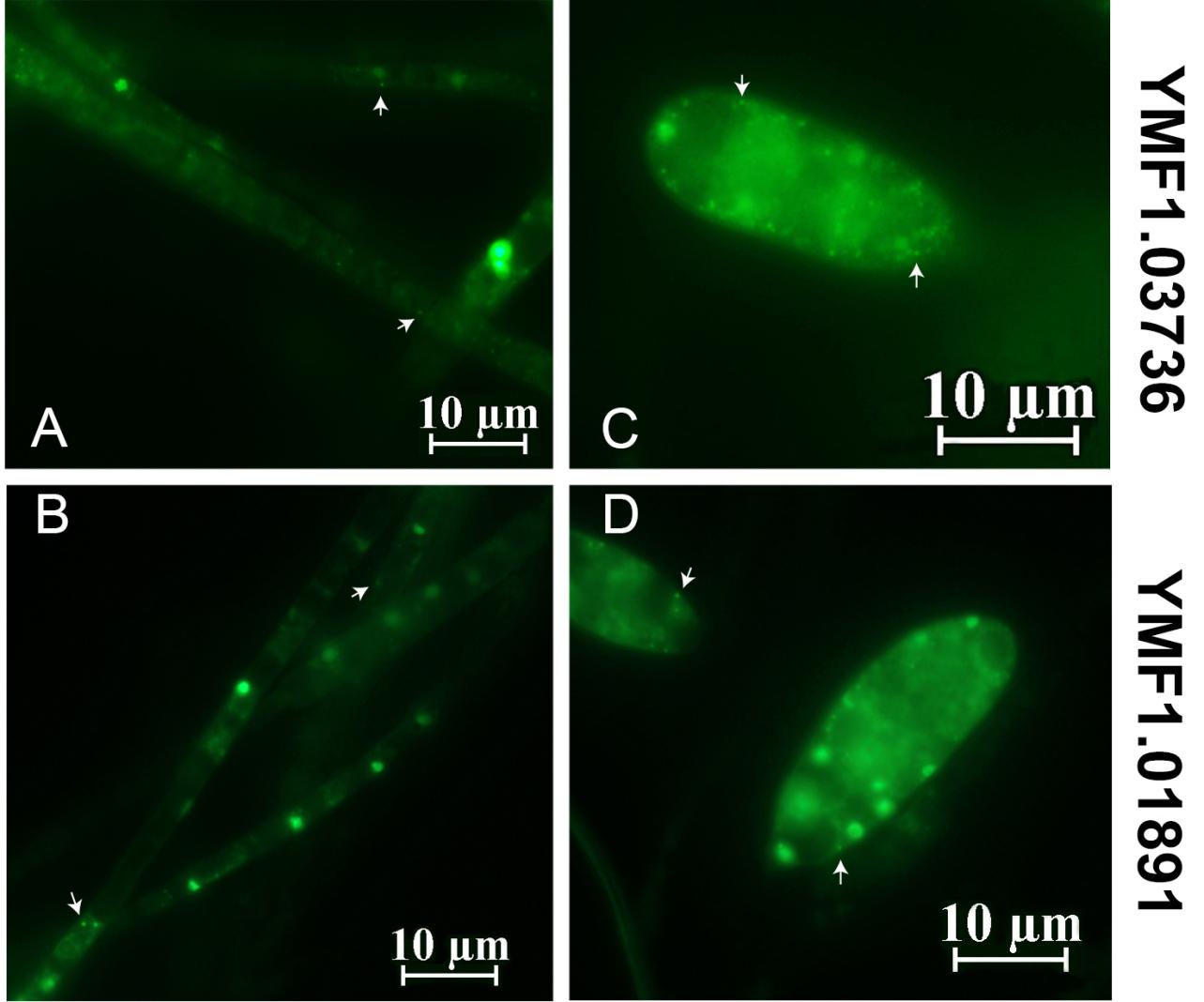
**

**Figure S2.** The detection of 16S rRNA in the total genomic DNA from *A. musiformis* cultures.

1−10 represent different *A. musiformis* strains, P represent a positive control.

**
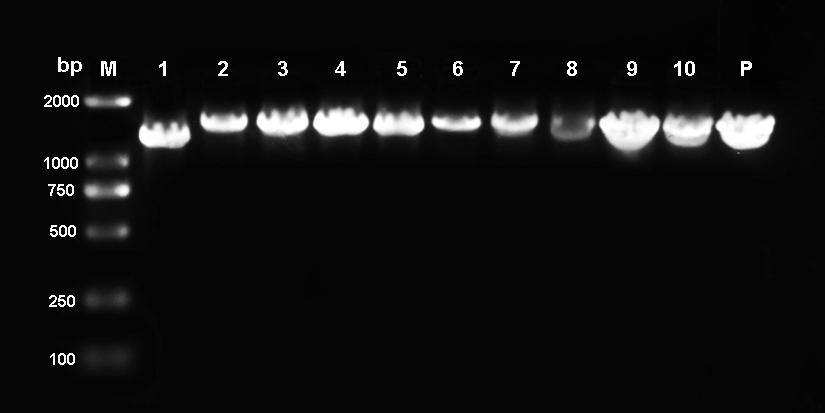
**

**Figure S3.** The rarefaction curves of 64 samples.

**
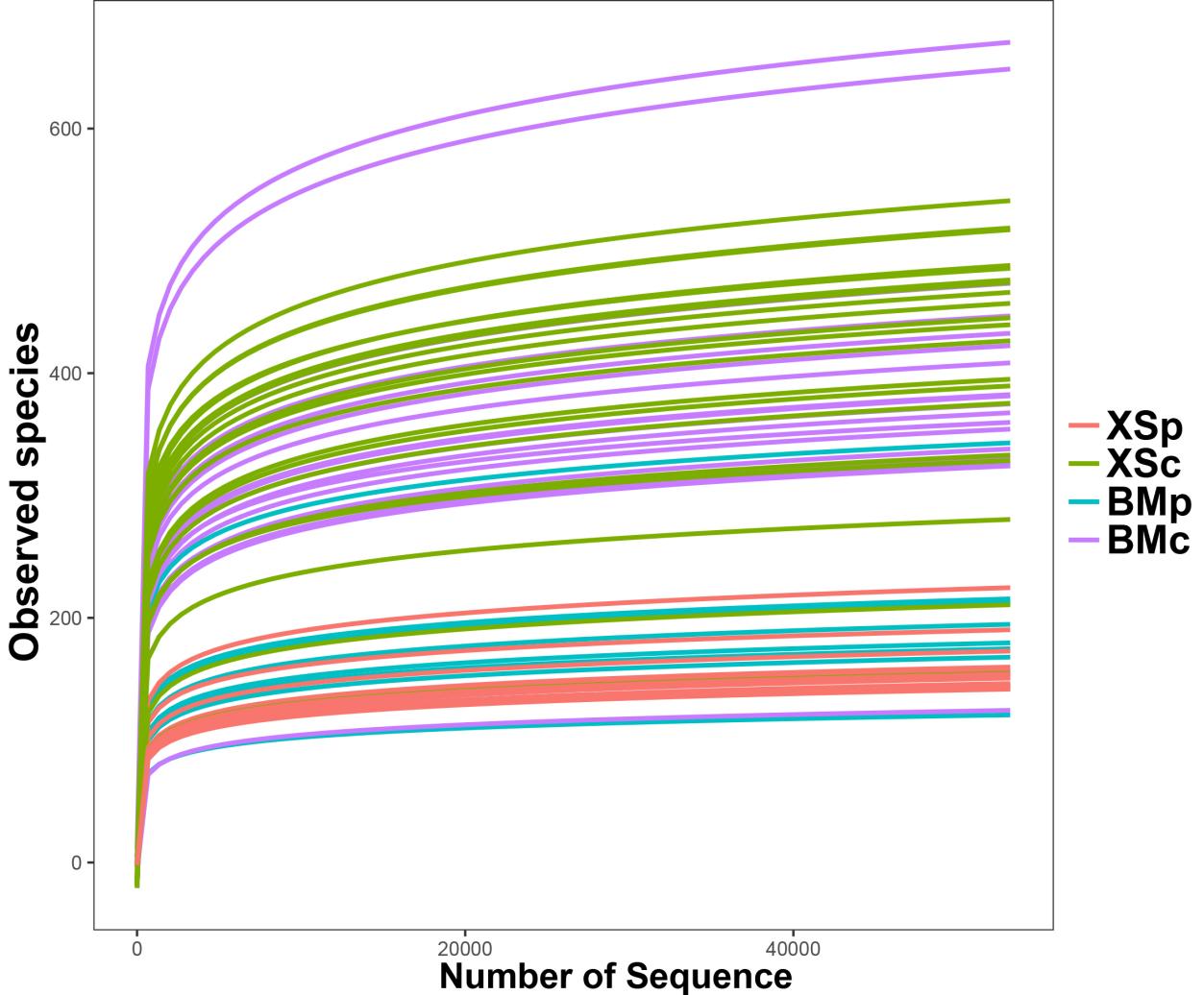
**

**Figure S4.** Venn diagram showing the unique and shared ASVs among different habitats and cultural conditions. (A) Samples from different habitats and cultured in PDB medium, (B) Samples from different habitats and cultured in CMB medium, (C) Samples from BM cultured in PDB and CMB medium, (D) Samples from XS cultured in PDB and CMB medium.

**
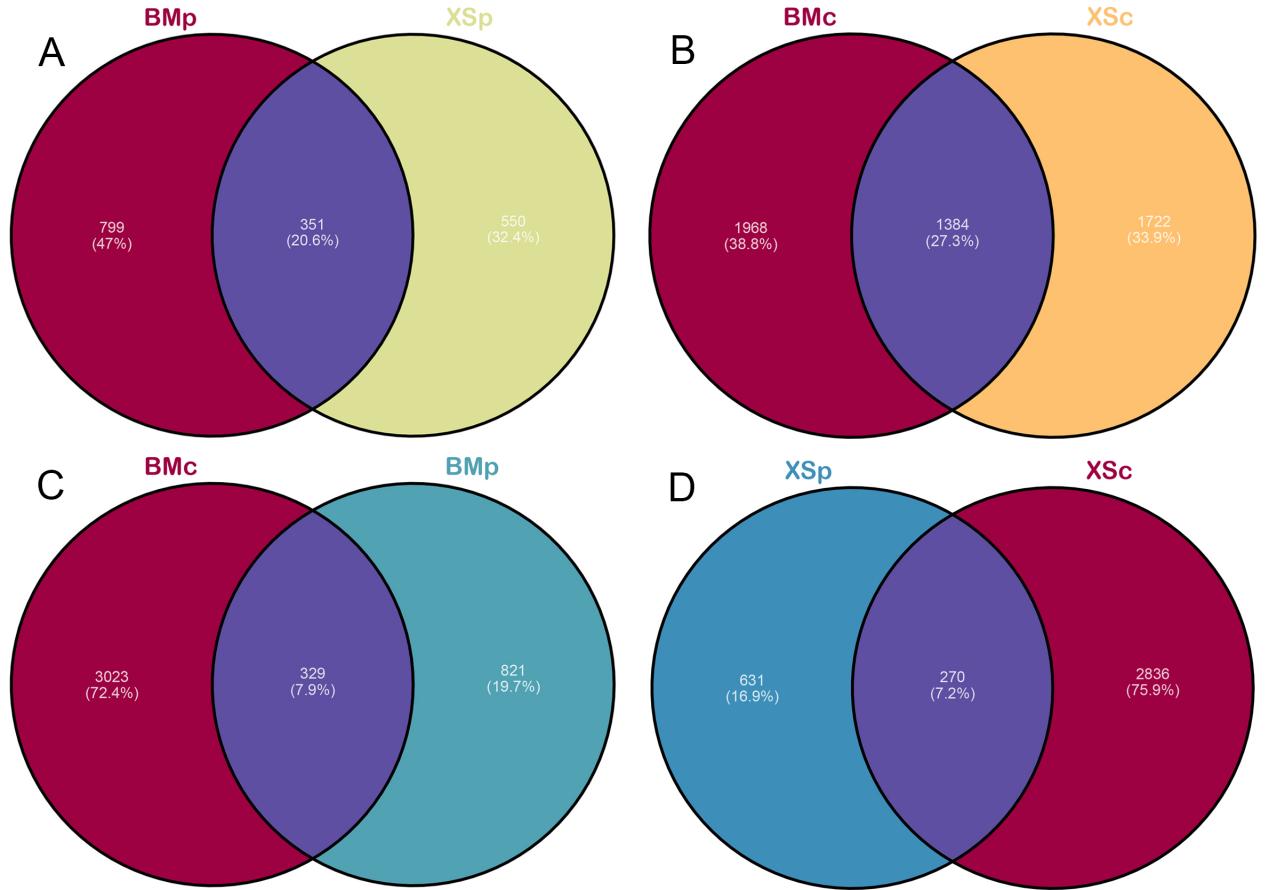
**

**Figure S5** Taxonomic composition of the whole bacterial microbiome in *A. musiformis* at phylum. level.


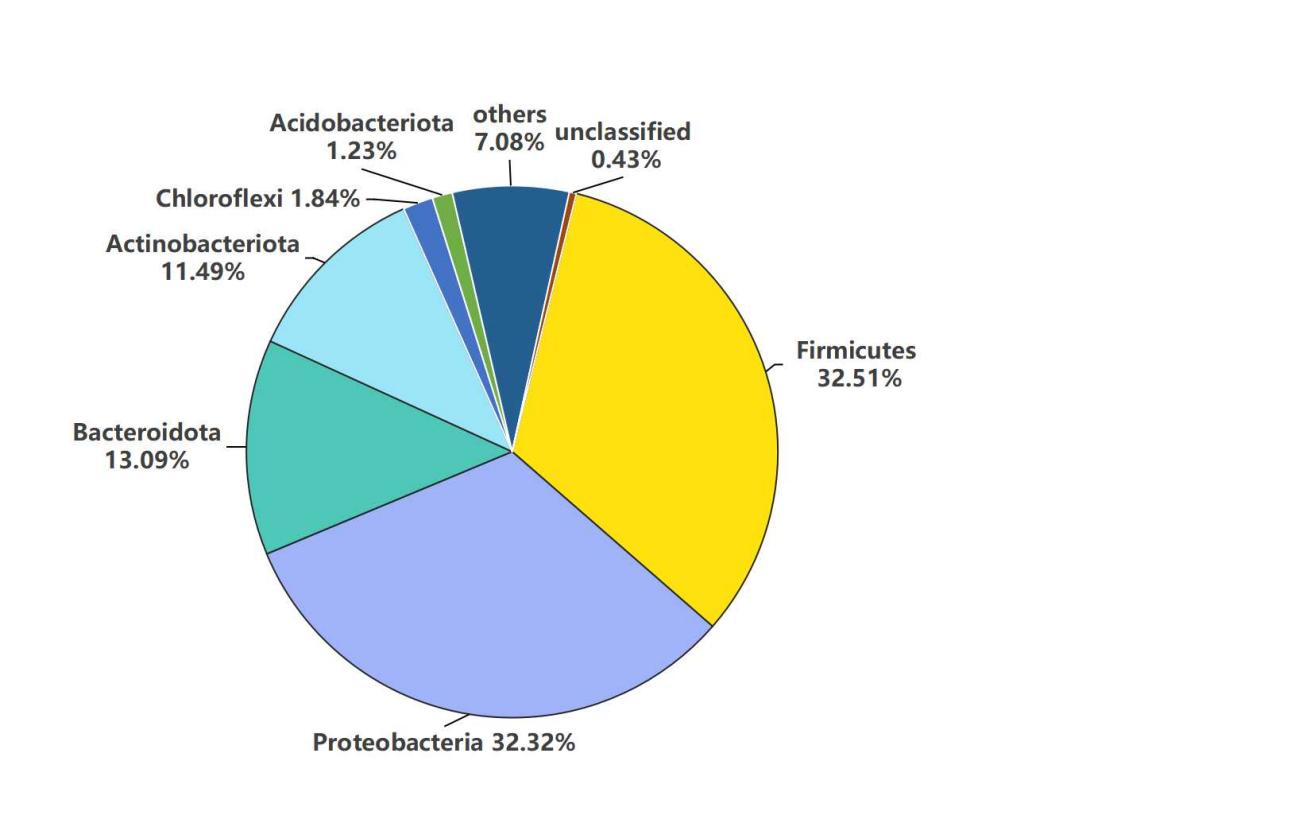


**Figure S6.** Heatmap displaying the abundance of differential nitrogen metabolism for the four groups (BMp, XSp, BMc, and XSc) in *A. musiformis*. The abundance of the differential N-cycling was predicted by FAPROTAX analysis.


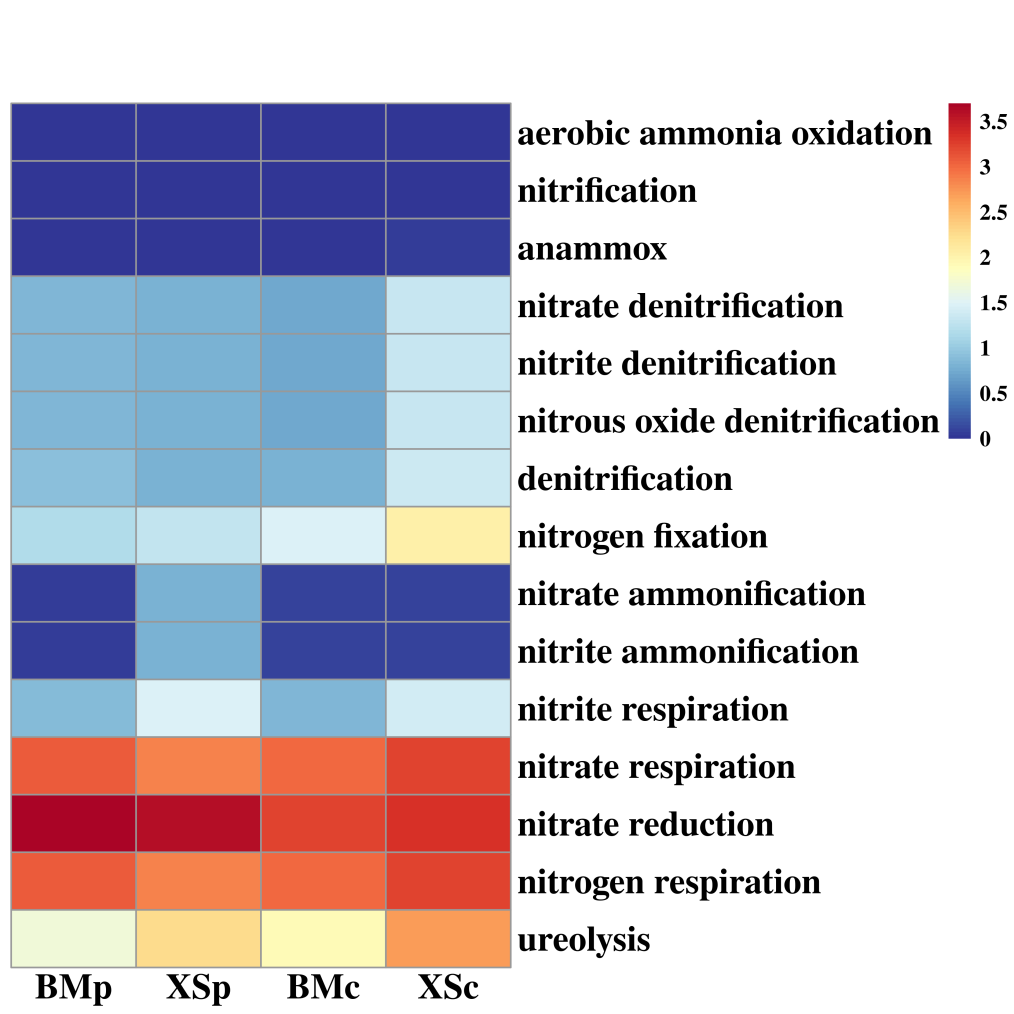


**Figure S7.** Phylogenetic tree of culturable ESB isolated from *A. musiformis* based on 16S rDNA. The blue sequences represented the type strain of the species.

**
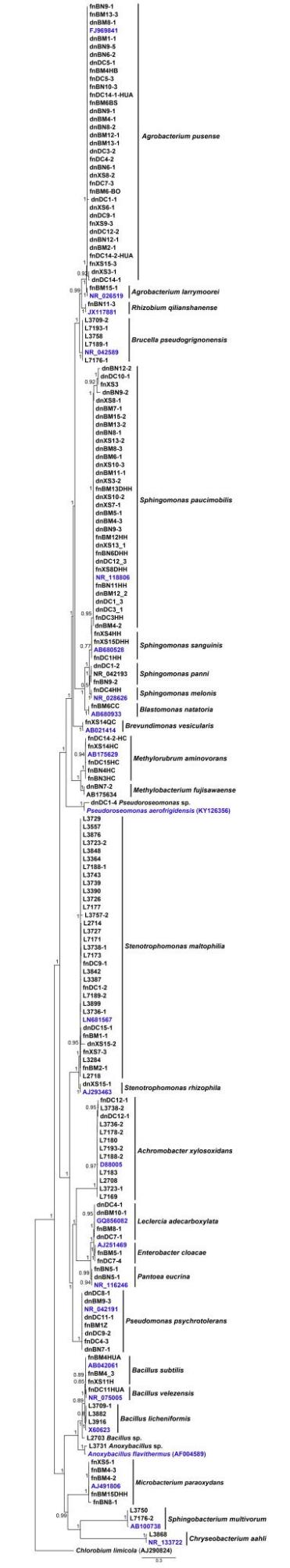
**

**Figure S8.** The effects of selected eight bacteria co-cultured with *A. musiformis* YMF1.07180. Sterile water treatment was used as a control (CK). BL: *Bacillus licheniformis*, AX: *Achromobacter xylosoxidans*, SM: *Stenotrophomonas maltophilia*, SP: *Sphingomonas paucimobilis*, AP: *Agrobacterium pusense*, PP: *Pseudomonas psychrotolerans*, LA: *Leclercia adecarboxylata*.

**
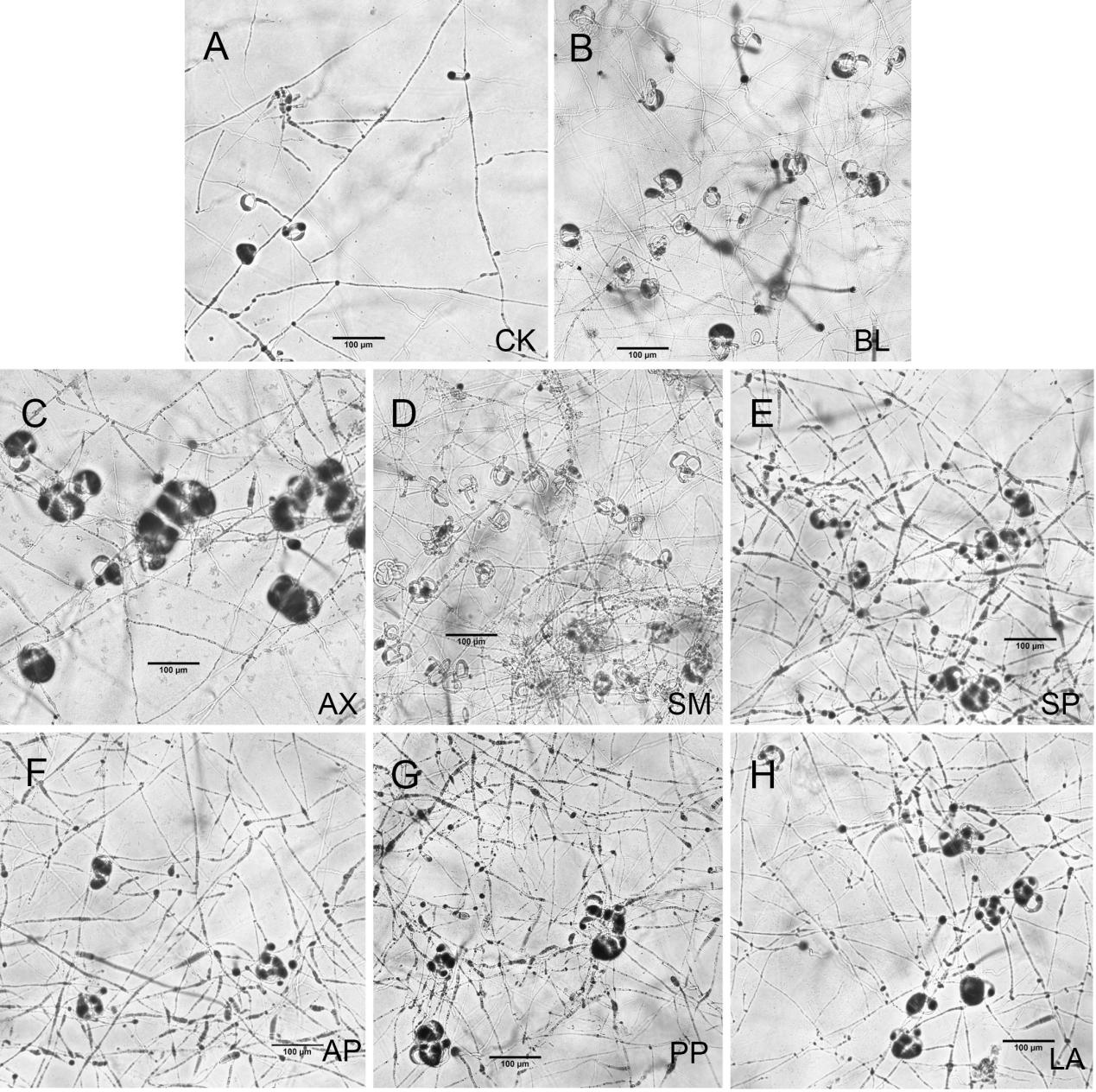
**

**Figure S9**. Prediction of the differential function of ESB in *A. musiformis* between different media (BM and XS indicate geographical origin, BMp and XSp indicate cultivation in PDB medium and BMc and XSc indicate cultivation in PDB medium) in KEGG categories at level 1 (A, D), level 2 (B, E) and level 3 (C, F). Relative signal intensity was normalized by the number of the genes for each indicated metabolic pathway. *, **, *** indicate the difference is at a significant level with *p* < 0.05, *p* < 0.01, and *p* < 0.001, respectively.


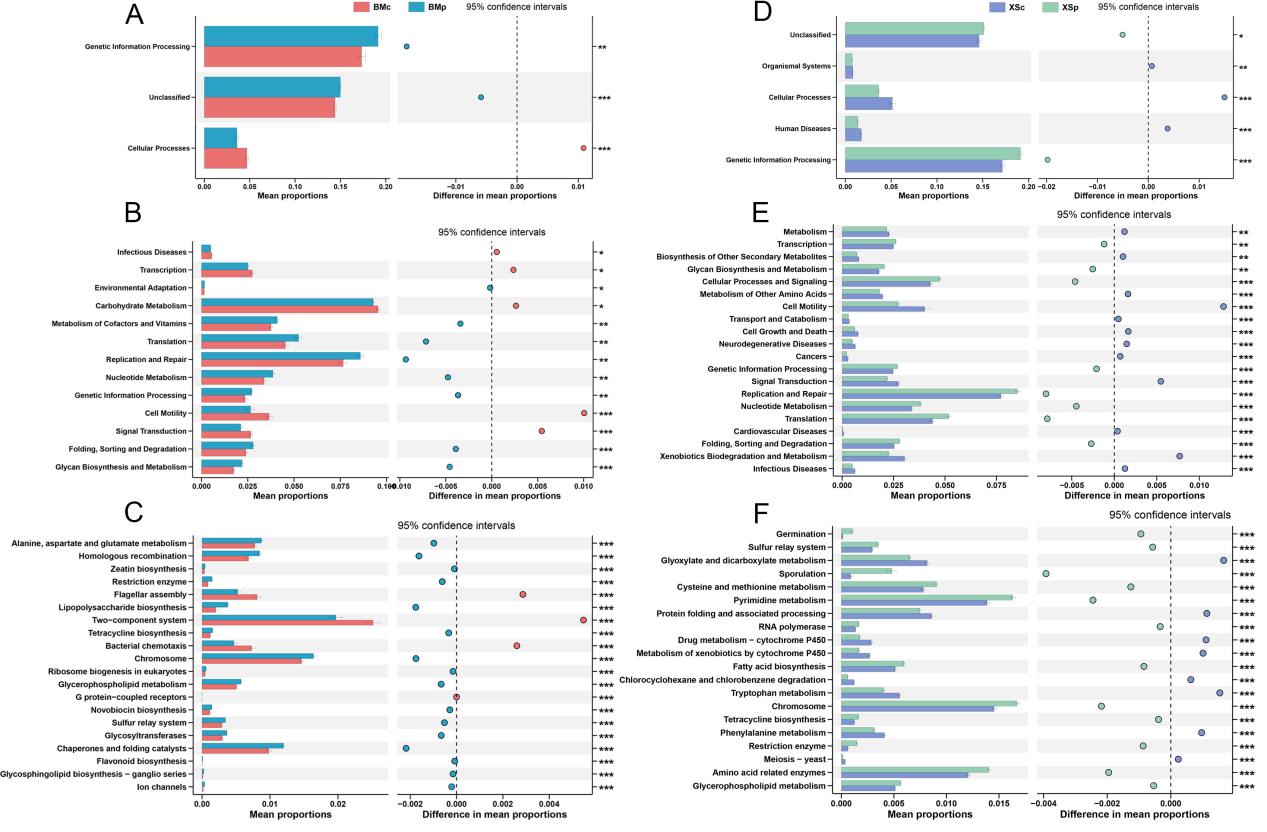

Supplement: Supplementary file 1 [file Data_Sheet_1.DOCX]
